# Competitive Carbonate Binding Hinders Electrochemical CO_2_ Reduction to CO on Cu Surfaces at Low Overpotentials

**DOI:** 10.1021/jacs.5c04518

**Published:** 2025-07-10

**Authors:** Jinhui Meng, Jessica Freeze, Linsey Nowack, Chaoyu Li, Hongsen Wang, Héctor D. Abruña, Adam P. Willard, Victor S. Batista, Tianquan Lian

**Affiliations:** † Department of Chemistry, 1371 Emory University, Atlanta, Georgia 30322, United States; ‡ Department of Chemistry and Energy Sciences Institute, 5755 Yale University, New Haven, Connecticut 06520, United States; § Department of Chemistry, 2167 Massachusetts Institute of Technology, 77 Massachusetts Avenue, Cambridge, Massachusetts 02139, United States; ∥ Department of Chemistry and Chemical Biology, 5922 Cornell University, Ithaca, New York 14853, United States

## Abstract

The electrochemical reduction of CO_2_ to useful chemicals holds promise for a sustainable carbon cycle. However, the key factors that control the pathways to various desired products remain unresolved, partially due to the limited knowledge of reaction intermediates on the electrode surface. To address this, we utilize in situ electrochemical shell-isolated nanoparticle-enhanced Raman spectroscopy in combination with density functional theory calculation to examine the potential-dependent composition of adsorbed species during CO_2_ reduction on polycrystalline copper. The results reveal that carbonate anion adsorption outcompetes other carbon-containing species, including adsorbed CO_2_ activation intermediate *COO^–^ and *CO, which has the effect of anodically shifting the onset potential of *CO formation in electrolyte solutions with a lower carbonate concentration. These results suggest that the competitive binding of carbonate impedes the reduction of CO_2_ on the Cu surface. Monte Carlo simulations show that both potential dependent electrode surface change and electrode-carbonate Coulomb interaction are key to understanding the competitive binding process. Our findings suggest that reducing the competitive binding of carbonate may be a promising route to improve the CO_2_ reduction on Cu electrodes at low overpotentials.

## Graphical abstract

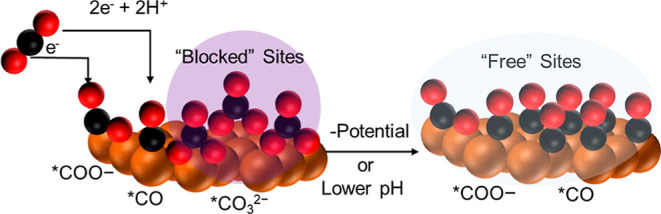

## Introduction

The electrocatalytic CO_2_ reduction reaction (CO_2_RR) has emerged as a promising solution for addressing the global challenge of greenhouse gas emissions, providing a way to store renewable electrical energy in the form of chemical bonds.^1,2^ Copper is a particularly promising electrode material for CO_2_ reduction due to its cost-effectiveness and its ability to generate energy-dense and economically valuable C_2+_ products (compounds with more than two carbons) with minimal overpotentials.^3–5^ For C_2+_ product-oriented pathways on Cu, *CO (asterisks indicate adsorption to the electrode) is a crucial precursor for C–C coupling reactions. A variety of theoretical, electrokinetic, and spectroscopic studies have been conducted on the C–C coupling reaction mechanism.^6–16^ In comparison, the initial stage of CO_2_ reduction on Cu, namely generating CO via CO_2_ activation is much less well understood.^6,17–19^

The reduction of CO_2_ to CO is a complex, multistep process that involves multiple intermediates and competing pathways,^1,20^ with an example shown in Scheme 1. Previous research has identified the reductive chemisorption of CO_2_ to a carboxylate group coordinated through a carbon and oxygen to the metal surface, η^2^(C,O)–COO^–^ (B in Scheme 1), as a possible intermediate for CO_2_ activation on Cu­(poly).^18^ It undergoes further reactions to produce either CO (D in Scheme 1) or formate, CHO_2_. However, the precise structure of the intermediates and their reaction pathways remains a subject of debate.^5^ For example, a strongly bound carboxylate intermediate (B in Scheme 1) can undergo protonation to form a carboxyl group, *COOH (C in Scheme 1), which can then be further reduced to make CO via proton-coupled electron transfer.^3,5^ Alternatively, a weakly bonded carboxylate can have its carbon protonated to form formyloxyl, *OCHO, which is further reduced to formate.^18^

**Scheme 1:**
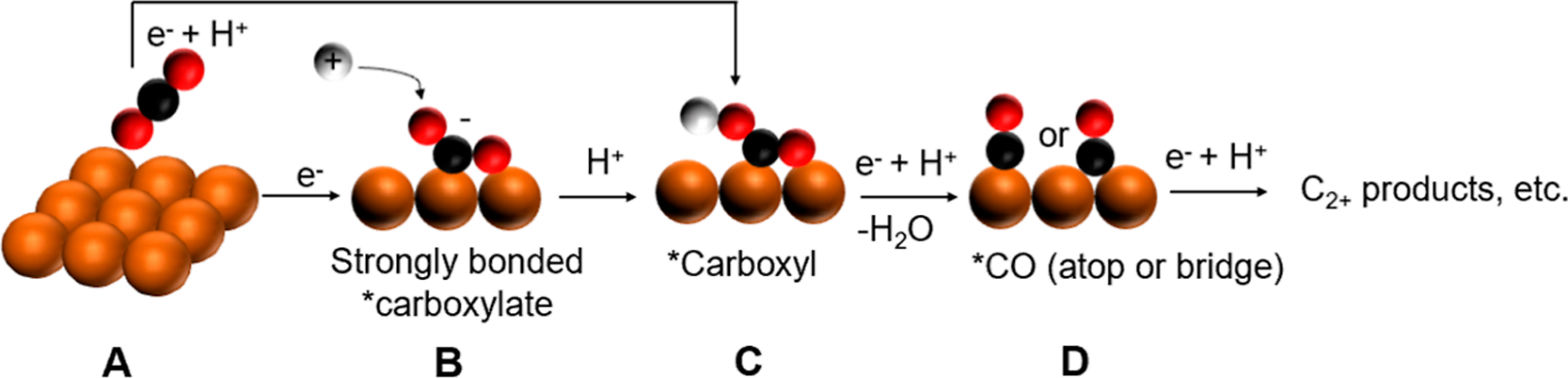
Pathway for the Electrochemical Reduction of CO_2_ to CO on Polycrystal Cu^a^

a Color code: carbon (black), oxygen (red), hydrogen (white), and copper (brown).

These and other proposed reaction mechanisms from CO_2_ to CO or formate^17,21,22^ have not explicitly accounted for the roles of other surface-binding species on a polarized Cu that can also affect activities. In a commonly used NaHCO_3_ aqueous solution at a polarized Cu interface, species such as carbonate,^23^ bicarbonate,^24,25^ Cu­(I) oxide/Cu,^6,17^ and *OH on Cu^15,19^ have been reported to alter the reaction through buffering, proton donor availability, and pH regulation. Deeper mechanistic understanding of these species is still lacking, due in part to the unclear assignments for the key surface intermediates and other unrevealed effects such as their specific binding competition. i.e., one inconsistent assignment is that the Raman peaks at ∼360 cm^–1^ and ∼1540 cm^–1^ appearing together at low overpotentials observed by multiple studies are usually assigned to different species, including bidentate adsorbed carbonate,^24,26–29^ a CO_2_-activated intermediate (cations stabilized adsorbed carboxylate, *COO ^–^M^+^),^18,30^ and a copper–carbonate-hydroxide intermediate (malachite).^31^

In this paper, we aim to provide a clear assignment on surface adsorbates during the CO_2_RR and study how carbonate/bicarbonate binding affects the CO_2_ to CO reduction reaction on polycrystalline Cu electrodes. We combine in situ shell-isolated nanoparticle enhanced Raman Spectroscopy (SHINERS) to identify key surface-bound intermediates. We assign these species with the help of density functional theory (DFT) calculations by considering the potential-dependent frequency shifts, isotope shifts, and binding energies under various reaction conditions. We examine the potential dependence of the population of key surface-adsorbed species, including *CO_3_^2–^, *COO^–^, and *CO, as a function of carbonate concentration in the electrolytes used for CO_2_ reduction to examine whether carbonate binds competitively with other species and how competitive binding affects the onset of *CO formation. To explore the physical origin of competitive binding, we perform Monte Carlo simulations of a lattice model of surface adsorption involving different species and compare them to additional control experiments of competitive binding between carbonate and CO. These results combined to reveal a revised CO_2_ reduction model, which explicitly considers the effects of carbonate competitive binding.

## Results and Discussion

### Raman Measurements

Figure 1A schematically shows the in situ SHINERS measurement for electrocatalysis on a polycrystalline Cu electrode in a three-electrode setup. Further information on the synthesis of nanoparticles, electrode preparation, electrochemical measurement, and Raman setup can be found in the Materials and Methods section and in Supporting Information-1. In this study, we use shell-isolated nanoparticles (SHINs) comprised of a 55 ± 5 nm diameter Au core and a 2–3 nm SiO_2_ shell (Figure S1), synthesized by a literature procedure.^32^ For operando SHINERS measurement, SHINS were deposited on the Cu electrode to form gap plasmonic modes that amplify Raman intensity by about 6 orders of magnitude (Figure 1A).^32^ The method has been successfully used for probing surface intermediates involved in CO/CO_2_ reduction to C_2+_ products on single and polycrystalline Cu electrodes.^10,33–36^

**Figure 1:**
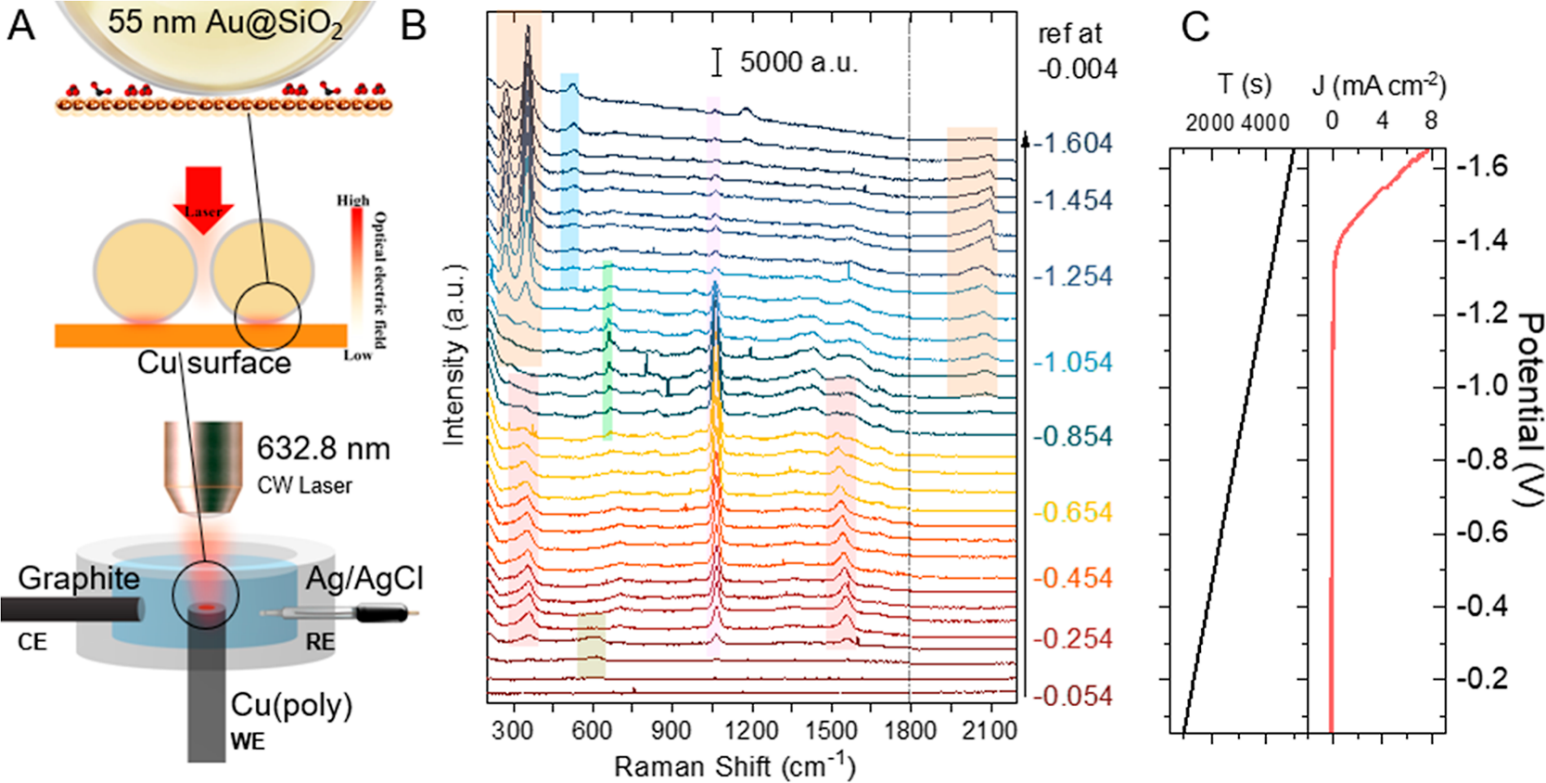
(A) Scheme of in situ electrochemical SHINERS measurement. (B) In situ Raman spectra of CO_2_ reduction to CO in 0.5 M NaDCO_3_/D_2_O on Cu­(poly) at indicated potentials (vs Ag/AgCl). Each potential indicates a range of +/25 mV. The same color-coded Raman peaks represent different modes of the same species. (C) Linear sweep voltammetry during spectra scan (potential applied as a function of time shown as the left black curve, obtained current density as a function of applied potential shown by the right red curve, scan rate 0.4 mV/s). The D-labeled electrolytes are used for clearer identification of species.

Figure 1B shows the representative potential-dependent in situ SHINERS difference spectra measured on polycrystalline Cu in a 0.5 M NaDCO_3_ D_2_O solution saturated with CO_2_. Spectra were obtained every 125 s during the linear sweep voltammetry (LSV) measurement from +0.346 V (vs Ag/AgCl) to −1.654 V at a 0.4 mV/s scan rate. Each spectrum is the average of a 50 mV potential range, and only the average potential is indicated in Figure 1B. The spectral windows from 100 to 1800 cm^–1^ and 1800 to 2200 cm^–1^ were measured separately and then combined together to generate the full spectrum. The represented linear sweep voltammograms (LSV) of these measurements are shown in Figure 1C. The raw spectra contain contributions of species within the double layer and in bulk solution, whose Raman spectra are dependent and independent of the applied potentials, respectively. To isolate the contribution of the former, all raw Raman spectra are subtracted from the reference (background) spectrum at −0.004 V to produce the corresponding difference Raman spectra, as shown in Figures 1B and S2–S4. The reference spectrum is chosen at −0.004 V, a potential at which no CO_2_ reduction-related reactions have occurred. Similar SHINERS spectra were also measured in H_2_O to help the assignment of OH/OD-related Raman peaks and have been analyzed in similar ways, as shown in Figures S3 and S4. From here on, only the subtracted spectra are shown. The potential-dependent Raman peaks in Figures 1B and 2 have been color-coded, with the modes of the same species in the same color, and can be attributed to surface-adsorbed species whose spectra (frequency and/or intensity) are affected by the change in the applied potential.

**Figure 2:**
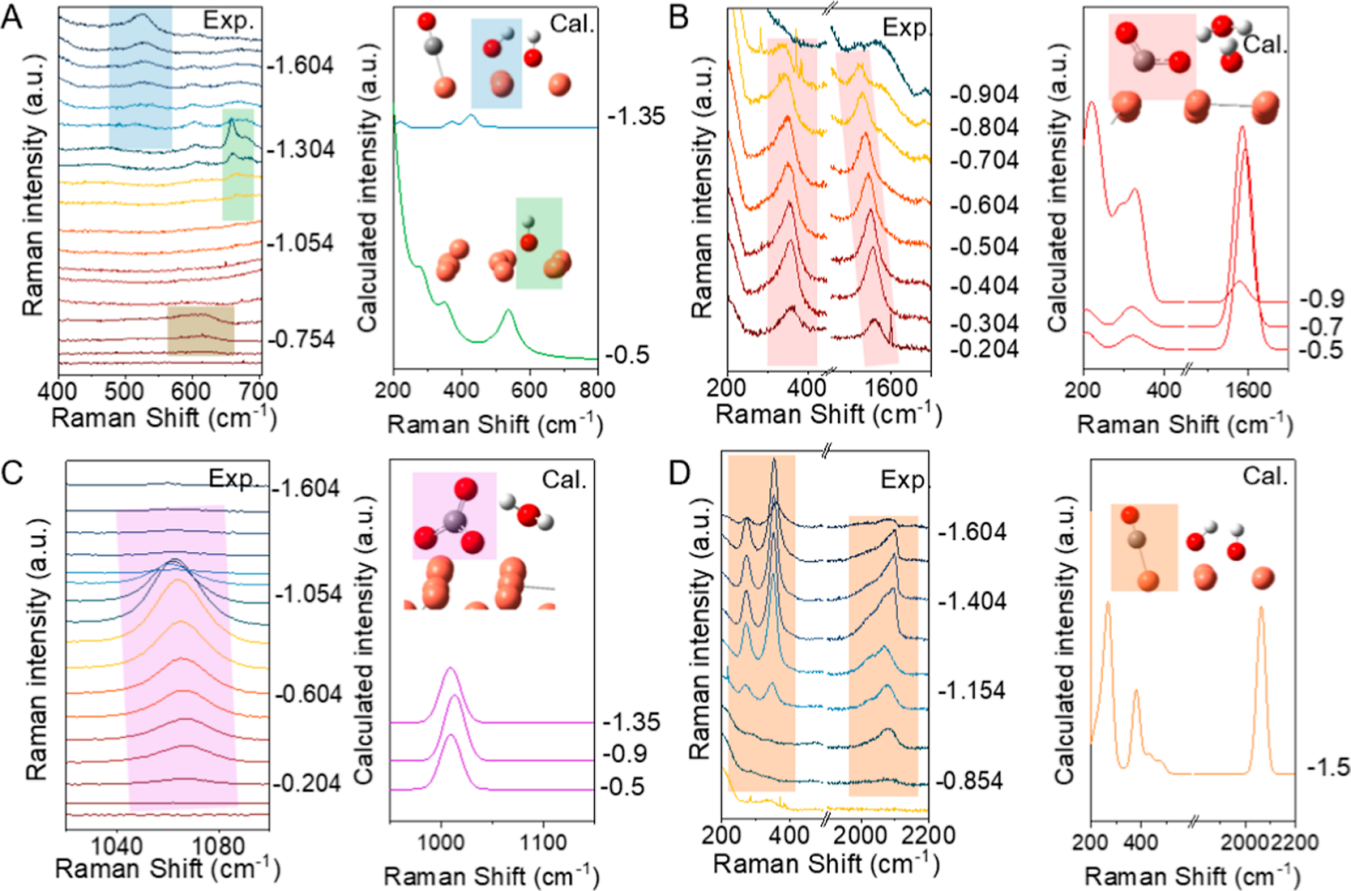
Comparison of measured potential-dependent Raman spectra (left) and computed Raman spectra (right) of proposed adsorbed species. Color codes for atoms: C (gray), O (red), D­(H) (white). (A) Cu–O species: Cu_2_O at 623 cm^–1^ (dark yellow shaded), Cu-OD at ∼ 670 cm^–1^ (green shaded), and CuO_*x*_(OD)_*y*_ at ∼525 cm^–1^ (blue shaded), (B) *COO^–^ M^+^ at ∼1540 and ∼340 cm^–1^ (red shaded), (C) *CO_3_^2–^ at ∼1070 cm^–1^ (purple shaded), (D) *CO at ∼280 cm^–1^, ∼360 cm^–1^, and 2080 cm^–1^ (orange shaded). Detailed H/D isotope effect and DFT calculations can be found in Supporting Information 2.2.

### Assignments of Raman Spectra

To assign the observed Raman spectral peaks, we conducted DFT calculations and compared the computed Raman spectra to the experimental observations in D_2_O. Figure 2A–D show the comparison of the measured and computed Raman spectra of key surface-adsorbed species and their computed structures, and additional computed spectra are provided in Figures S5–S8. We used three key observations to facilitate the assignment: Raman frequencies, the direction of potential-dependent vibrational frequency shifts (Stark shifts), and H/D isotopic effects (Table S1 and Figures S9–S15) and the detailed assignment and discussion are provided in Supporting Information Sections 2.3–2.5. The observed surface species can be classified into two distinct categories: carbon-containing species (*CO, *COO^–^, and *CO_3_^2–^) and noncarbon-containing (Cu oxygen related) species. The ∼1540 cm^–1^ and the ∼360 cm^–1^ peaks from −0.2 V to −0.85 V (red shaded in Figure 2B) are assigned to surface cation stabilized carboxylate (*COO^–^ M^+^). This assignment is consistent with a recent report,^18^ although different assignments have also been reported.^24,26–29,31^ Detailed discussion of this assignment is provided in Supporting Information 2.4. The Raman peak at ∼1055 cm^–1^ from −0.2 V to −1.3 V (purple shaded in Figure 2C) is assigned to surface-adsorbed carbonate anions (*CO_3_^2–^), following literature reports and the carbonate peak in solution.^37,38^ The Raman peaks at ∼2080 cm^–1^, ∼290 cm^–1^, and ∼350 cm^–1^ from −0.904 V to −1.604 V (orange shaded in Figure 2B) are assigned to the surface adsorbed carbon monoxide (*CO), consistent with literature reports. The Raman peak at ∼623 cm^–1^ from −0.054 to −0.204 V (dark yellow shaded in Figure 2A) can be assigned to Cu_2_O following literature reports.^10,39^ The ∼660 cm^–1^ peak and ∼523 cm^–1^ peak (green and blue shaded in Figure 2A) are tentatively assigned to Cu–O-D­(H) species, as discussed in Supporting Information 2.5. Both their spectral assignments and roles in the CO_2_ reduction reaction remain unclear and will not be further discussed in this work. Table S1 summarizes the assignments of the key species along with their corresponding vibrational modes.

### Potential Dependent Adsorbate Coverage during CO_2_ Reduction

Using the surface species assignments in the previous section, Figure 3A plots each adsorbate’s normalized Raman intensity as a function of applied potential during a cathodic scan of a Cu electrode in CO_2_-saturated 0.5 M NaHCO_3_ solution (the plot including more species under the same condition can be found in Figure S16). This figure illustrates an interchange between electrolyte anions and the CO_2_ reduction intermediates on the surface. We divided the overall interchange into a sequence of four stages in the cathodic scan. In Stage 1 (Cu_2_O reduction), from ∼0.0 to −0.3 V, Cu_2_O is reduced to Cu, following eq 1, which initiates surface coadsorption of *COO^–^ and *CO_3_^2–^, following eqs 2a and 2b, respectively. These processes lead to the onset of a small Faradaic current shown in Figure 3A lower panel. This result suggests that poly-Cu reduction (to a Cu-rich surface) is required before CO_2_ reduction can proceed, consistent with previous reports.^10,13,39,40^ In Stage 2 (competitive binding), from ∼−0.3 to −0.85 V, the surface coverage of *CO_3_^2–^ continues to increase, while that of *COO^–^ reaches a peak value and then decreases. At around −0.85 V, the Cu surface is primarily covered by *CO_3_^2–^. As discussed in the next section, this is attributed to the strong binding of carbonate to Cu, which inhibits the formation of more *COO^–^. In Stage 3 (carbonate desorption), from −0.85 V to −1.4 V, the increasingly negative surface charges caused by applied potential lead to desorption of *CO_3_^2–^, eq 2b, which coincides with the formation of *CO, a product of CO_2_ reduction, following eq 3. Over this potential range, *CO, instead of *COO^–^ is a stable intermediate. This observation is also consistent with the competitive binding of *CO and *CO_3_^2–^ reported previously^28,29,41,42^ In Stage 4 (CO reduction), from −1.4 to −1.6 V, the population of *CO decreases while the Faradaic current increases, indicating an increasing rate of *CO reduction.

1 $${Cu}_{2}O+{2e}^{-}+{2H}^{+}⇌Cu+H_{2}O$$

2a +*CO2+e−⇌COO−*

2b +*CO32−⇌CO32−*

3 COO−*+e−+H+⇌CO*(staticallystable)+OH−

**Figure 3:**
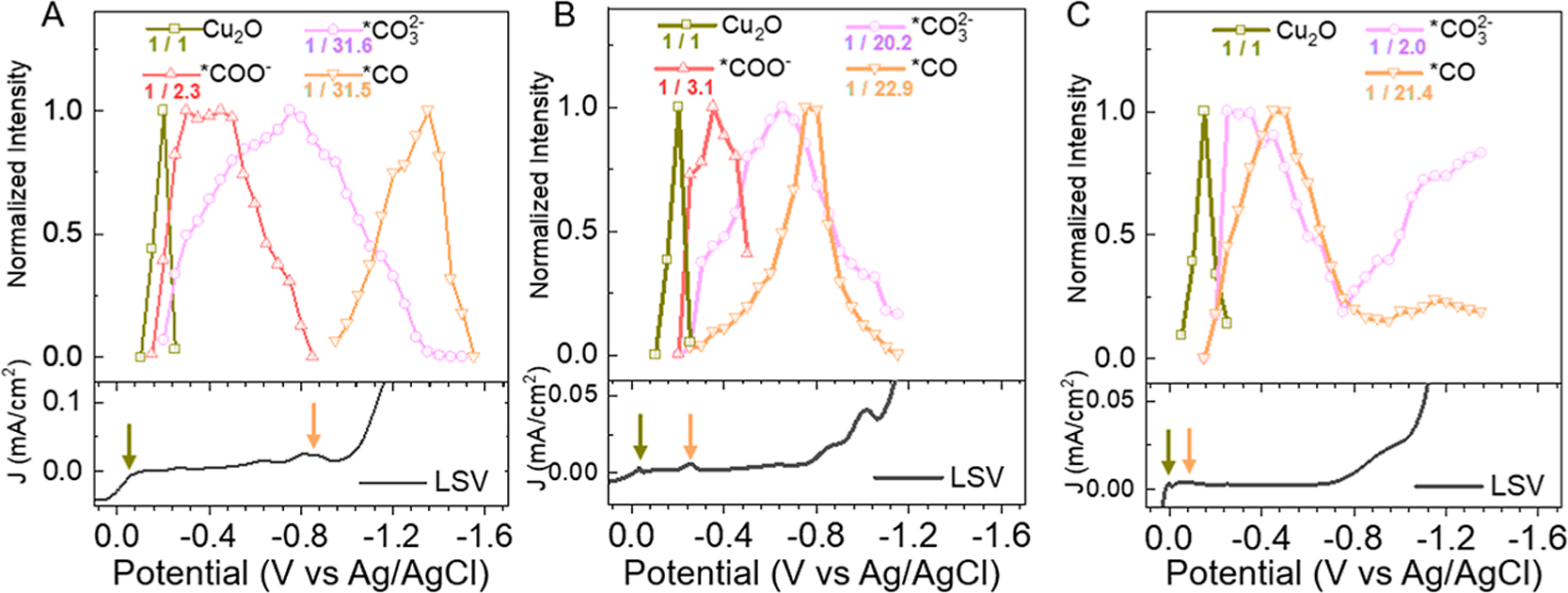
Potential dependent surface population change during CO_2_ reduction (top) and linear sweep voltammetry current density (bottom) in electrolyte (A) E1 (0.5 M NaHCO_3_, saturated with CO_2_, pH = 7.2), (B) E2 (0.1 M NaHCO_3_ + 0.4 M NaClO_4_, saturated with CO_2_, pH = 6.8) and (C) E3 (0.5 M NaClO_4_, saturated with CO_2_, pH = 4.6). Scan rate 0.4 mV/s. Raman frequency of key species: 623 cm^–1^ (Cu_2_O, gray squares), 1540 cm^–1^ (*COO^–^, red triangles), 1065 cm^–1^ (*CO_3_^2–^, purple circles), ∼2000–2100 cm^–1^ (*CO, orange inverted triangles). The intensity was normalized to the maximum intensity of Cu_2_O and the scaling factors are labeled in the legend (e.g., 1/2.3 indicates the original intensity values are scaled down by 2.3 times). The color arrows in LSV panels represent the onset potentials for the species plotted in the same color in the top panels. Relevant Raman spectra for B and C can be found in Supporting Information 2.8 (Figures S17 and S18).

To test this hypothesis of carbonate competitive binding with *COO^–^ and *CO, we carried out a study of the effect of carbonate concentration on the CO_2_ reduction. This is done by comparing three electrolyte solutions: (E1) CO_2_-saturated 0.5 M NaHCO_3_ (pH = 7.2), (E2) CO_2_-saturated 0.1 M NaHCO_3_ + 0.4 M NaClO_4_ solution (pH = 6.8), and (E3) CO_2_-saturated 0.5 M NaClO_4_ solution (pH = 4.6). The result of E1 has been shown in Figures 1, 2, and 3A and discussed above. These solutions have different ratios of NaHCO_3_ and NaClO_4_, but the total ion concentrations are kept the same to maintain the same ionic strength. Perchlorate is chosen for this experiment because perchlorate anions are reported to be nonadsorbing species on Cu electrodes.^28,41^ Because carbonate is in equilibrium with bicarbonate in solution, its concentration is controlled by the concentration of initially added bicarbonate (specified above). The equilibrium also dictates that changing carbonate concentration is necessarily accompanied by a change of solution pH. Thus, both the effects of pH and carbonate concentration changes on CO_2_ reduction should be considered.

The potential dependent Raman spectra during the cathodic scan in electrolyte solutions E2 and E3 are shown in Figures S17 and S18, respectively. Figure 3A–C compares the normalized intensity of key species as a function of potential for the three electrolytes of various bicarbonate concentrations. SHINER intensity depends on the population, Raman cross section, and field enhancement factor, and because the field enhancement factor varies from one measurement to another, this variation should be accounted for to compare relative populations from different measurements. To do that, the highest intensity of Cu_2_O is set to one, and all other species are normalized relative to Cu_2_O for each measurement in Figure 3A–C, and the normalization factors, which set the peak relative intensity to 1, are indicated in the legend. If we assume that the Cu_2_O signal is proportional to the electrode surface area, the normalization factor indicates the relative peak population of these species in different electrolyte solutions per unit surface area. As shown in Figure 3A–C, the relative peak *CO_3_^2–^ coverage is 31.6, 20.2, and 2.0 in electrolytes E1, E2, and E3, decreasing at lower initial bicarbonate concentrations in the electrolyte. Although in electrolyte E3, no carbonate or bicarbonate was added to the solution, a small amount of carbonate can be expected due to conversion between dissolved CO_2_, carbonic acid, bicarbonate, and carbonate in CO_2_-saturated solution.^43^ Furthermore, the potential of peak *CO_3_^2–^ coverage shifts from ∼−0.8 V in E1 to ∼−0.6 V in E2 and ∼−0.2 V in E3. As shown in Figure S21, the estimated potential of zero charge (PZC) of Cu electrodes is at ∼−0.85 V, ∼−0.4 V, and ∼−0.15 V in electrolytes E1, E2, and E3, respectively, which correlates with potentials of peak *CO_3_^2–^ coverage. *CO onset occurs at ∼−0.85 in electrolyte E1 (Figure 3A), ∼−0.25 V in E2 (Figure 3B), and ∼−0.15 V in E3 (Figure 3C), shifting to more anodic potentials in electrolytes with lower bicarbonate concentration. We also plot the *CO intensity under the RHE scale in Figure S20, which also shows a similar trend in the *CO onset potentials in these electrolytes. The LSV responses in these electrolyte solutions, shown in the lower panel of Figure 3 and detailed in Figure S19B, indicate peaks at −0.3 V in E2 and −0.1 V in E3, in accordance with the increasingly greater anode onset potentials of *CO peak in the Raman spectra. We also conducted DEMS measurements to support the observation of earlier CO formation (seen in Supporting Information 2.10), but bulk phase CO was below the instrument detection limit to be detected. It should be noted that similar potential dependent peak intensity changes of *CO, *CO_3_^2–^ have been reported previously and were discussed according to a different mechanism,^31,44^ which is discussed in Supporting Information 2.9.

The existing potential window for *COO^–^, we believe, has its stability strongly influenced by the pH of the local environment. From E1 to E3, the system becomes increasingly acidic, and under lower pH conditions, protons more readily react with *COO^–^ to form *COOH. Since *COOH is a highly reactive and short-lived intermediate, it is rapidly converted to *CO once formed. As a result, in E3, we no longer observe spectral signatures of either *COO^–^ or *COOH. The change in *CO formation onset potential in electrolytes E1, E2, and E3 may be caused by different pH and/or different *CO_3_^2–^ coverage (competitive binding). Previous experimental studies^45–47^ have shown that for CO_2_ to CO reaction at the potential range where CO is the major product, neither the onset potential of CO detected by DEMS nor the experimental current due to CO_2_RR to CO exhibit significant pH dependence, and a zero reaction order for protons was found in all possible proton sources (HA,^45^ bicarbonate,^48^ water^49^) in the electrolytes. The independence of the CO onset potential on the pH was rationalized by a model with the formation of *COO^–^ being the rate-determining step (RDS) in the CO production at low overpotentials (specifically around −0.2 V in our case). Thus, we attribute the earlier formation of *CO in E2 and E3 to anodic shift of the *CO_3_^2–^ competitive binding (or “poisoning”) on the surface, which frees up active sites for CO_2_ reduction. According to the *CO_3_^2–^ competitive binding model, under low carbonate binding conditions (both in E2 and E3), the onset of *CO formation begins when Cu_2_O reduction occurs (∼−0.15 V). In contrast, under high carbonate binding conditions (E1 and E2), *CO formation is delayed due to the much stronger adsorption of *CO_3_^2–^ that blocks surface reaction sites.

### Competitive Carbonate and CO Binding

During the CO_2_ reduction study described above, the potential dependent competitive binding of *CO_3_^2–^ with *COO^–^ and *CO is convoluted with the potential dependent CO_2_ reduction reaction itself, which hinders quantitative modeling. To better understand the competitive binding process, we carried out a study of competitive binding between *CO_3_^2–^ and *CO in CO-saturated carbonate solution using both in situ Raman spectroscopy and Monte Carlos (MC) modeling. This study involves Raman measurement in three electrolytes: (1) the first experiment probes the potential dependent adsorption behavior of *CO_3_^2–^ in the absence of CO in C1 electrolyte (0.25 M Na_2_CO_3_ aqueous solution saturated with Ar, pH = 11.95); (2) the second experiment investigates the potential dependent adsorption of *CO on Cu in the absence of carbonate in C2 electrolyte (0.5 M NaClO_4_ aqueous solution saturated with CO, pH = 7.62); and (3) the third control experiment examines the competitive binding of *CO_3_^2–^ and *CO in C3 electrolyte (0.5 M NaHCO_3_ aqueous solution saturated with CO, pH = 8.44).

To examine the potential dependent *CO_3_^2–^ in the absence of CO or CO_2_ reduction intermediates (*CO, *COO^–^), we carried out in situ SHINER measurement in C1 electrolyte (Ar-saturated 0.25 M Na_2_CO_3_, pH = 11.95). The Raman spectra (shown in Figure S25) during a cathodic scan from +0.1 V to −1.8 V show a clear *CO_3_^2–^ peak. The spectra show negligible signatures associated with *COO^–^ and *CO (∼360 cm^–1^, ∼1540 cm^–1^, and ∼2080 cm^–1^), which is consistent with the negligible amount of dissolved CO_2_ and associated CO_2_ reduction intermediates under the alkaline pH conditions in an Ar-saturated solution. Therefore, under these conditions, the potential dependent *CO_3_^2–^ intensity reflects the direct competitive binding between carbonate anions and water on Cu surfaces (without the participation of other carbon-containing species). The normalized intensity of *CO_3_^2–^ and MC simulated population of *CO_3_^2–^ as a function of potential is plotted in Figure 4A, both of which show a peak at around −0.8 V and decreases at more positive or negative potentials. This potential dependent *CO_3_^2–^ intensity profile in Ar (pH ∼ 12.0, Figure 4A) resembles that in CO_2_-saturated 0.5 M NaHCO_3_ solution (pH ∼ 7.2, Figure 3), despite the fact that their peak potentials vary from −0.86 V in Ar to −0.75 V under the CO_2_RR (shown in Supporting Information 2.9). The similarity suggests that CO_2_ and CO_2_ reduction intermediates (*COO^–^ and *CO) have a negligible effect on the surface population of *CO_3_^2–^, which is likely determined by its competitive binding with water. In addition, because of the large pH difference in these solutions, this similarity also suggests that the potential-dependent *CO_3_^2–^ Raman intensity profile is not caused by a potential-dependent local pH change near the Cu electrode surface.

**Figure 4:**
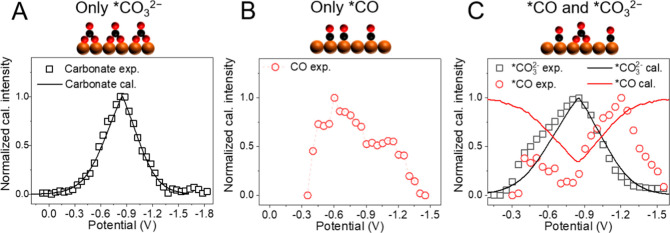
Experimental and Monte Carlo modeling of competitive binding between *CO_3_^2–^, *CO and H_2_O on Cu electrodes. (A) Comparison of potential dependent coverage of *CO_3_^2–^ in the absence of *CO measured by SHINER in C1 electrolyte (0.25 M Na_2_CO_3_ aqueous solution saturated with Ar, pH = 11.95) (hollow square) and calculated by MC simulation (solid black line). (B) Potential dependent coverage of *CO in the absence of *CO_3_^2–^ measured by SHINER in C2 electrolyte (0.5 M NaClO_4_ aqueous solution saturated with CO, pH = 7.62). (C) Comparison of potential dependent coverage of *CO_3_^2–^ and *CO measured by SHINER in C3 electrolyte (0.5 M NaHCO_3_ aqueous solution saturated with CO, pH = 8.44) and simulated by MC modeling. Black squares and red circles represent the measured *CO_3_^2–^ and *CO results, and black and red solid lines represent the MC simulated *CO_3_^2–^ and *CO coverages, respectively. Relevant Raman spectra can be found in Supporting Information 2.11 (Figures S25–S28).

To investigate how *CO population on Cu changes with potential in the absence of *CO_3_^2–^, we measured the SHINER spectra of *CO on Cu in a C2 electrolyte (CO-saturated 0.5 M NaClO_4_, pH = 7.62). Perchlorate solution is chosen as a control since it has been shown to be a weak binder to Cu among other anions.^28,41^ The raw Raman spectra during cathodic scan from +0.046 to −1.504 V (Figures S25) show a peak of ClO_4_^–^ ions (∼946 cm^–1^) and *CO (∼2080 cm^–1^). The ClO_4_^–^ peak shows negligible potential dependent frequency and intensity (Figure S26 and S27), suggesting the signal is dominated by ClO_4_^–^ ions in the bulk solution with negligible interaction with the electrode. The *CO peak frequency and intensity show a pronounced potential dependence. *CO peak appears at −0.3 V, near the potential of Cu_2_O reduction; increases its intensity at more negative potential to reach a maximum at ∼−0.7 V (Figure 4B) and decreases in intensity at further cathodic scan due to the reduction of CO (see Supporting Information 2.9 Figure S19 and Supporting Information 2.11 Figure S26 for details). The computed *CO population is not shown in Figure 4B because the experimentally observed volcano-shaped *CO profile arises from a complex interplay of processes, including the gradual electrochemical reduction of Cu_2_O to metallic Cu and the reduction consumption of *CO to form further products. These processes are difficult to capture accurately in computational models.

To examine how *CO adsorption competes with *CO_3_^2–^, we carried out a Raman measurement in C3 electrolyte (CO-saturated 0.5 M NaHCO_3_ aqueous solution, pH = 8.44). Figure 4C shows the corresponding experimental Raman intensities and calculated populations for *CO_3_^2–^ and *CO. Figure 4C reveals a similar symmetric profile for adsorbed carbonate’s intensity that shows a peak at ∼−0.85 V. This feature is the same as those in the absence of CO or CO_2_ (Figure 4A) and under CO_2_ reduction conditions (Figure 3A), which indicates that the characteristic potential-dependent surface coverage of carbonate is not altered by the presence of carbon-containing species (*CO, *COO^–^, *CO_2_). Moreover, *CO intensity exhibits a bimodal curve with the local minimum coinciding with the potential at which carbonate’s intensity maximizes. This is clear evidence of competitive adsorption between *CO_3_^2–^ and *CO. During the cathodic scan, *CO intensity shows the first maximum at −0.35 V, suggesting that at low surface coverage (before −0.35 V), both *CO_3_^2–^ and *CO coverage increases at more negative potential, until *CO_3_^2–^ outcompetes and displaces *CO at −0.35 V to ∼−0.8 V. At more negative potential, *CO_3_^2–^ desorption occurs, and *CO coverage increases until reaching the second peak coverage at −1.2 V, after which *CO coverage decreases, likely due to the reduction of CO, similar to that observed in the absence of carbonate (Figure 4B) and under CO_2_ reduction conditions (Figure 3A).

To better understand the consequences of competitive binding of *CO_3_^2–^ with the carbon-containing species observed above, we carried out Monte Carlo simulations. In these simulations, adsorption interactions are modeled as a sum of two contributions: an intrinsic binding energy and a potential-dependent surface coulomb energy. More specific details of the simulation are provided in Supporting Information 2.13, and only the key findings are summarized here. The potential dependent *CO_3_^2–^ surface coverage in the absence of carbon-containing species, as shown in Figure 4A, can be modeled by the competitive binding of *CO_3_^2–^ and *H_2_O. At potentials negative of the PZC, one expects that Coulombic repulsion between the anion and a negatively charged electrode will promote *CO_3_^2–^ desorption, increasingly so at more negative potentials. This effect is captured in our Monte Carlo model where, at negative potentials relative to the PZC, water gradually replaces *CO_3_^2–^ on the lattice (Figure 4A). The potential dependent *CO_3_^2–^ coverage at potentials positive for the PZC is more complicated. Based on Coulombic effects, one expects that at more positive potentials, *CO_3_^2–^ will be increasingly attracted to the surface until it saturates the available binding sites and its intensity plateaus. Since during a cathodic scan the *CO_3_^2–^ Raman intensity turns on after Cu_2_O reduction and increases gradually as the surface becomes less positively charged (toward PZC), it may indicate a slow potential-dependent surface rearrangement that weakens carbonate binding at positive potentials. This hypothesis is supported by a rapid intensity decrease of carbonate at −0.15 V, the potential of Cu oxidation in an anodic scan (Figure S29), and the large hysteresis of *CO_3_^2–^ in the cathodic and anodic scans. In our Monte Carlo model, we represent this effect on *CO_3_^2–^ by giving the adsorbate a V-shaped intrinsic binding energy (Figure S30), where it is most stable at the experimental maximum Raman intensity. This empirical V-shaped binding energy in our Monte Carlo model is difficult to physically justify yet essential to replicating the results of the next section.

In the presence of CO, as shown in Figure 4C, the Monte Carlo model includes the competitive binding of *CO_3_^2–^, *CO, and *H_2_O. The simulation shows that *CO coverage minimizes at the potential with maximal *CO_3_^2–^ coverage, and recovers its population on either side of the *CO_3_^2–^ peak (Figure 4C). Our modeling results are in qualitative agreement with experimental results, and they support the assumption that carbonate dominates adsorption competition over other CO-related species at potentials around −0.7 V. Unlike the experiment, however, our model does not include Cu oxidation/reduction events. Consequently, the simulated *CO population does not decrease from −0.3 to −0.5 V like it does in the experiments. Most importantly, the *CO intensity in this system displays a bimodal profile, which is similar to the sum of *COO^–^ and *CO species under CO_2_ reduction (−0.9 V, Figure 3A), This suggests that strongly adsorbed carbonate outcompetes other carbon-containing adsorbates.

### New Competitive Binding Model for CO_2_ Reduction

Our results show that *CO_3_^2–^ outcompetes *COO^–^ and/or *CO over the potential range of −0.3 to −1.0 V (vs Ag/AgCl). A similar competitive binding of *CO_3_^2–^ and *CO has been observed previously in CO-saturated bicarbonate solution in the range of −0.56 V–1.05 V vs SHE (−0.78–0.83 V vs Ag/AgCl).^29^ Thus, we propose that *CO_3_^2–^ competes with many carbon-containing intermediates on the Cu surface, including *CO_2_, *COO^–^, *COOH, and *CO, and this competitive binding delays the onset of CO_2_ reduction by blocking the surface reaction sites. CO_2_ reduction can only occur when *CO_3_^2–^ desorbs from the surface at a negative potential. The conventional CO_2_ reduction pathway shown in Scheme 1 does not fully capture this important aspect of the reaction mechanism. We propose a revised reaction mechanism in Scheme 2, in which the competitive binding step is explicitly considered. Scheme 2 depicts three key species involved in three representative potentials for a CO_2_ reduction process on Cu electrodes in a CO_2_-saturated 0.5 M NaHCO_3_ electrolyte. At −0.15 V, where most Cu_2_O is reduced to Cu, CO_2_ and CO_3_^2–^ coadsorb on the surface to form *COO^–^ and *CO_3_^2–^. At −0.7 V, *CO_3_^2–^ outcompetes *COO^–^ in binding and dominates the surface, decreasing the number of available surface sites for CO_2_ activation. Finally, at −1.2 V, the surface coverage of *CO_3_^2–^ decreases, which opens up surface sites for the conversion of CO_2_ to CO (with *CO on the surface as a product).

**Scheme 2:**
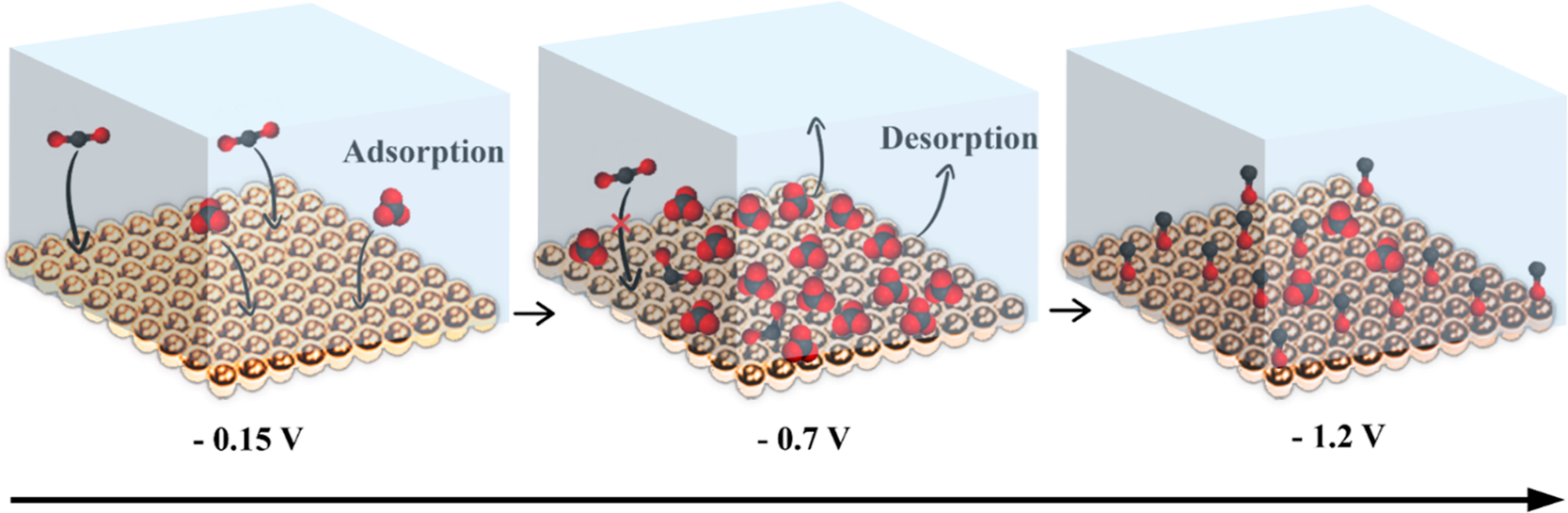
Competitive Binding Model for CO_2_ Reduction^a^

a Schematic representation of potential-dependent surface species during CO_2_ activation at selected potentials along a cathodic scan in the low overpotentials region for a Cu­(pc) electrode in a CO_2_-saturated 0.5 M NaHCO_3_ electrolyte. Only major species are shown: CO_2_, CO_3_^2–^, and CO. Color code for atoms: Cu atoms (brown), C atoms (black), O atoms (red), and aqueous electrolyte (the blue cube on top of the surface). The species shown represent the static and stable intermediates detected on Cu surfaces under steady-state conditions. Rapid steps that are not rate-limiting, such as proton transfer, are not depicted in this scheme.

Thus, we believe that at least three key factors are essential to the onset of *CO generation from CO_2_ reduction on Cu. First, a relatively Cu-rich surface (from Cu_2_O reduction) to enable the stable adsorption of carbon-containing species; second, a favorable local environment with enough proton sources and sufficient electrochemical potential to enable CO_2_ reduction (CO pathway); third, available surface sites without electrolyte competitive binding, such as carbonate in our case. It is also reasonable to infer that the diminished carbonate binding in a more acidic medium may be one of the reasons to explain the different CO_2_ reduction selectivity in acidic electrolytes.^45,50^ Especially on Cu, lowering pH not only affects the possible pathways and energy parameters,^51^ but also depletes the surface adsorbed carbonate. Therefore, the selection of less-surface-binding electrolytes can also be a parameter in improving the CO_2_ reduction reaction. It is important to note that the electrolyte binding strength also varies for different metals. For example, on Au electrodes, no competitive binding between *CO_3_^2–^ and *CO is observed,^29^ and when pH is tuned from 7.4 to 5.7 with the same ionic strength, the onset potential for the detection of bulk CO production also does not change.^52^

## Conclusions

In summary, we conducted a combined spectroscopic and computational study of the effects of the competitive binding of carbonate anions on CO_2_ reduction on polycrystalline copper electrodes at the low overpotentials region. First, we used in situ surface-enhanced Raman spectroscopy and potential-dependent DFT calculation to identify adsorbed species on Cu, including *CO_3_^2–^, *COO^–^ M^+^, *CO, and Cu-hydroxyls. We show that the Raman peaks at 1540 cm^–1^ and 360 cm^–1^ can be assigned to *COO^–^M^+^, a key intermediate of the CO_2_ activation. The potential dependence of Cu_2_O, *CO_3_^2–^, *COO^–^M^+^, and *CO surface coverage suggests that Cu_2_O reduction is required for a CO_2_ reduction reaction to occur, and there is competitive binding between *CO_3_^2–^ and other C-containing species, including *COO^–^M^+^ and *CO. Comparison of potential dependent surface coverage of key species in electrolytes with different carbonate concentrations shows that, at lower carbonate concentrations, the onset of *CO formation shifts to more anodic potentials, suggesting that competitive carbonate binding delays the onset of CO_2_ reduction to CO. Further control experiments of carbonate and CO binding suggest that near or at the more positive of the potential of zero charge of the Cu electrode, carbonate binding is much stronger than *CO and *COO^–^M^+^ and this can be understood through MC simulation to be caused by the strong electrostatic interaction between the natively charged carbonate and the positively charged electrode. We combined these findings to propose a new CO_2_ reduction mechanism in which the competitive electrolyte, especially carbonate, binding is explicitly considered. In short, our findings suggest that reducing competitive carbonate binding will be an important consideration for improving the CO_2_ reduction on Cu electrodes.

## Materials and Methods

### Chemicals and Materials

Na_2_CO_3_ (99.999%) and NaH^13^CO_3_ (99%) were purchased from Acros Organics and Cambridge Isotope Laboratories, respectively. All electrolytes were prepared by using ultrapure water (Milli-Q, 18.2 MΩ·cm). Deuterium labeling experiments were prepared with D_2_O (99.9 atom % D) from Sigma-Aldrich. NaClO_4_ (hydrate, 99.99%) was purchased from Sigma-Aldrich. To prepare the CO_2_-saturated NaHCO_3_ solutions, the Na_2_CO_3_ solutions were purged with high purity CO_2_ gas (99.99%, nexAir) overnight. Ar- and N_2_-saturated solutions (Na_2_CO_3_, NaClO_4_) were prepared by purging the prepared solutions for 2 h with ultrahigh-purity Ar (99.999%, nexAir) or N_2_ gas (99.999%, nexAir). CO solutions were purged under ultrahigh purity CO (99.999%, PRAXAIR) for 2 h, A pH meter (Accumet basic AB15) was used to measure the pH of all electrolytes prior to use.

### Electrode Preparation

Polycrystalline Cu disc electrodes (ALS Co., Ltd., representative from CH Instrument in the US) with an OD of 6 mm were used as working electrodes. To prepare a smooth working electrode, the Cu working electrode was polished on a micro cloth disk with the slurry of 0.3, 0.1, and 0.05 μm alumina separately. Then, the electrode was sonicated with ultrapure water and methanol for 3 min each. After that, the Cu electrode was rinsed by ultrapure water and electrochemically cleaned in 85% H_3_PO_4_ at 1.5 V vs Ag/AgCl (1 M KCl) for 30 s. Then, the working electrodes were rinsed with copious amounts of ultrapure water before assembling with SHINs. 1–2 μL of diluted as-prepared SHiNs was drop cast on cleaned Cu electrodes, and the solvent was allowed to vaporize under continued N_2_ gas flow before usage. Electrochemical measurements, DEMS measurements, and preparation and characterization of SHiNs are described elsewhere in Supporting Information.

### In Situ Raman Measurements

SHiNs solution was first prepared and then drop-cast onto the polished Cu disk electrode (diameter = 3 mm, CH Instrument) and fixed in an electrochemical spectroscopic Raman cell with graphite as a counter electrode and Ag/AgCl (1 M KCl) as a reference electrode. The cell was sealed after electrode fixation and electrolyte injection. An electrochemical workstation (CHI1205c, CH instrument) was then connected with the cell. For spectroscopic measurements, a home-built Raman system was used (Figure S2). The system utilizes a He–Ne laser (632.8 nm, Thorlabs) as the light source and objectives (10× and 50×) for signal collection. An average laser power from 1.2 to 4.7 mW was used to avoid damage or interference by light on samples during the measurements. The signal collection system includes a spectrograph (Shamrock, Andor) and an electron-multiplied charge couple device (EMCCD) (Newton, Andor).

### DFT Calculation Details

Calculations of chemical species on Cu surfaces were conducted using Gaussian 16, version C.01,^53^ with optimizations and frequency calculations performed to tight convergence using the unrestricted ωB97XD^54^ functional, which contains dispersion, the 6-31+G­(d,p)^55–69^ basis set for atom types [N, C, O, H, Na], and LANL2DZ^64,65^ for Cu atoms. All systems were computed in water using the Solvation Model Based on Density (SMD)^54^ with a Solvent Accessible Surface (SAS)^67^ surface description. Ultrafine integer grids were used, and reorientation of symmetry by the algorithm was disabled to maintain consistent field direction among all calculations. The field was defined as being normal to the Cu surface. Field to experimental potential was determined based on the potential of zero charge being treated as equivalent to a calculation with no field, followed by calculations of positive and negative fields being systematically increased and decreased until an experimental match was found. This was done due to limitations on the understanding of surface coverage and thickness by the adsorbates that greatly inhibit the direct use of field-to-potential conversion equations.^67,68^

Cu­(100) and Cu(111) surfaces were explored for the adsorption of reaction intermediates by comparison of spectral peaks in calculated versus experimental spectra. Each Cu surface was formed as a two-layer-deep 4 × 4 block according to the facet organization and by utilizing the Atomic Simulation Environment (ASE)^70^ python library with a lattice constant of 3.597 Å.^70^ Copper atoms, except those directly interacting with adsorbates, were frozen for optimization. Careful starting placement of adsorbates was done to prevent edge placements that may describe unrealistic surfaces. A potential of zero charge was chosen based on the previous experimental reports.^71^

### Monte Carlo Calculation Details

The competitive adsorption on Cu(100) between relevant CO_2_ reduction species is modeled with Monte Carlo (MC) simulations. In this model, the change in energy for species *i* to adsorb to a copper site is represented as

$$\Delta E_{i}=\Delta E_{PZC,i}+\alpha_{i}q_{i}\left(\varphi +0.9\right)$$

where the first term (Δ*E*_PZC,*i*_) is the intrinsic binding energy of adsorbate *i* to Cu at the potential of zero charge (PZC). The second term is the Coulombic interaction of charge (*q*_*i*_) of adsorbate *i* with the electrode surface under an electrical potential (φ). The potential energy is shifted to be zero at the experimentally determined potential of zero charge (−0.9 V vs Ag/AgCl). The second reflects the work to bring a charged adsorbate from the electrolyte bulk to the electrode surface. A constant, α_*i*_, scales this work and may be used to account for other potential-dependent interactions and heterogeneity on the surface. The Metropolis-Hastings Algorithm is used to sample the surface ensemble, where the acceptance ratio for the change *A + *B* → *B + *A* is

$$p_{A\to B}=\frac{m_{B}}{m_{A}}e^{-\left(\Delta E_{B}-\Delta E_{A}\right)/k_{b}T}$$

with *m*_*i*_ being the molality of *i* in the bulk, *k*_b_ being the Boltzmann constant, and *T* being the temperature. The lattice size for a simulation is 200 × 200 sites, and simulations were run for 2 × 10^6^ steps with analysis done on the last 10% of the simulation. See Supporting Information for additional simulation details.

## Supplementary Material



## Notes

Extended description of experimental methods (materials preparation, electrochemical SHINERS setup, and Raman and DEMS measurements); in-depth analysis and discussion of the key conclusions based on extra experimental and computational spectra data (Raman background subtraction, DFT-calculated Raman spectra, peak assignments, and *COO^–^/Cu–OH species identification), as well as the reasoning for ruling out other possible assignments and the interpretation of the data; and potential-dependent spectral changes, *CO_3_^2–^ population analysis, Cu_2_O reduction-induced carbonate intensity variations, MC simulation results, and estimates of potentials of zero charge (PDF)
