# Supplementary material for: Competitive Carbonate Binding Hinders Electrochemical CO2 Reduction to CO on Cu Surfaces at Low Overpotentials
Source: J Am Chem Soc. 2025 Jul 10;147(29):25361–71. doi: 10.1021/jacs.5c04518 (PMC12291447; doi:10.1021/jacs.5c04518)
Supplement: Supplementary file 1 [file ja5c04518_si_001.pdf]

Supplementary Materials for

Competitive Carbonate Binding Hinders Electrochemical CO_2 Reduction to CO on Cu Surfaces at Low Overpotentials

Jinhui Meng[1], Jessica Freeze[2], Linsey Nowack[3], Chaoyu Li[1], Hongsen Wang[4], Héctor D. Abruña[4], Adam P. Willard[3], Victor S. Batista[2*], and Tianquan Lian[1*]

[1]Department of Chemistry, Emory University, Atlanta, Georgia 30322, United States;[2]Department of Chemistry and Energy Sciences Institute, Yale University, New Haven, Connecticut, 06520, United States; [3]Department of Chemistry, Massachusetts Institute of Technology, 77 Massachusetts Avenue, Cambridge, Massachusetts 02139, United States; [4]Department of Chemistry and Chemical Biology, Cornell University, Ithaca, New York 14853, United States.

This file includes:

Supplementary text

Figs. S1 – S30

Tables S1 - S3

References

Contents

1. Extended description of experimental methods 3

1.1 Additional materials preparation 3

1.2 Electrochemical cell for SHINERS measurement 3

1.3 Raman measurements 4

1.4 DEMS measurements 4

2. Extended results, analysis and discussion 5

2.1 Discussion on the background subtraction of Raman spectra 5

2.2 Additional DFT calculated Raman spectra of key species 8

2.3 Summary of the Raman peaks assignment 9

2.4 Additional discussion on *COO$^-$ peaks assignments 13

2.5 Additional discussion on Cu-OH related species assignments 18

2.6 Supplement electrochemical Raman spectra on *CO 21

2.7 Additional plot of potential dependent intensity 21

2.8 Additional results on *CO_3^{2-} population dependence 22

2.9 Estimation of potentials of zero charge for systems 24

2.12 Discussion on Cu_2O reduction induced carbonate intensity change 31

2.13 Additional MC simulation results 32

2.14 Additional control results 37

3. References 39

1. Extended description of experimental methods

1.1 Additional materials preparation

Preparation of nanoparticles. Au and Au@SiO_2 nanoparticles were prepared based on the methods developed by Li, et al[1]. The obtained shell-isolated nanoparticles were characterized by TEM and electrochemical methods to verify the SiO_2 shell fully covered the Au core.

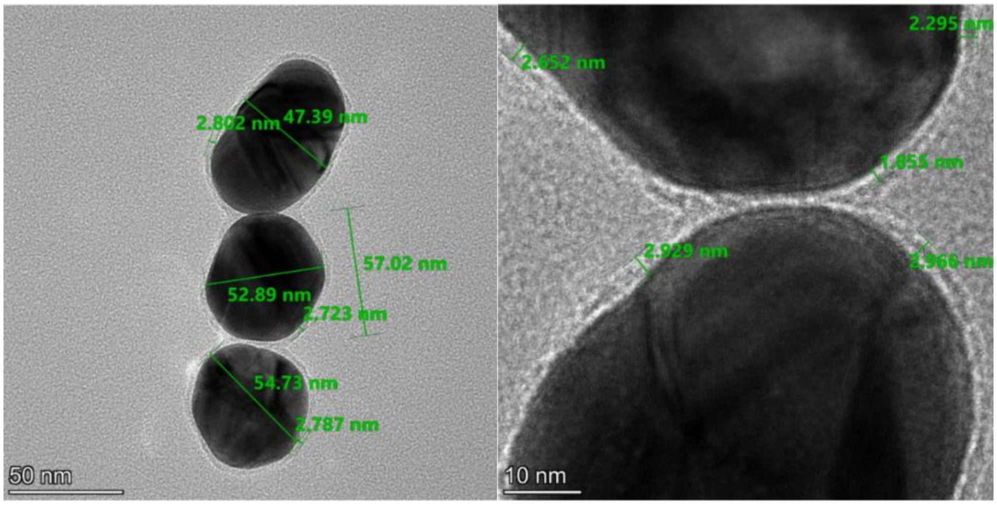

Figure S1. High resolution TEM image of synthesized Au@SiO_2 nanoparticles. diameter ~55 nm, thickness of Si_2O shell ~ 2-3 nm.

Preparation of Cu working electrodes. The polycrystalline Cu disk electrodes (OD 6 mm, ALS, Co., Ltd) were firstly polished with 0.05 μm Al_2O_3 with polishing pads, then rinsed with ultrapure water and electrochemically cleaned in 85% H_3PO_4 at 1.5 V vs. Ag/AgCl (1 M KCl) for 30 seconds until showing mirror-like surfaces. The functionalization of SHiNs (Au@SiO_2 nanoparticles.) is then performed by drop-casting the droplets of pre-saturated and washed NP solutions on the Cu electrode, the electrode is then put under dry air to vaporize the solvent water and the NPs are left on electrode surface.

1.2 Electrochemical cell for SHINERS measurement

A home-built 3-electrode Teflon electrochemical cell was used in this work (Figure 1 A). A potentiostat (CH Instruments) was used to control the potential. The electrochemical cell body was cleaned in boiling mixed concentrated acids (H_2SO_4/HNO_3, 1:1) for one hour followed by

sonication and thorough rinse with ultrapure water before use. An Ag/AgCl electrode (1 M KCl, CH Instruments) was used as a reference electrode. The potentials were converted to the reversible hydrogen electrode (RHE) scale using E(RHE) = E(Ag/AgCl) + 0.0591×pH + 0.236. A graphite rod was used as a counter electrode respectively, to eliminate possible contamination from the Pt counter electrode, all spectroscopic electrochemical measurements were reproduced with the graphite rod counter electrode. The distance between the electrode surface and the cell window is estimated to be ~ 250 μm.

1.3 Raman measurements

A home-built Raman system was utilized for all Raman measurements in this work. The system includes a polarized 632.8 nm He-Ne laser source, corresponding needed optics, and Raman signal was collected by a spectrograph (Shamrock, Andor) and further detected by an electron-multiplied charge coupled device (Newton EMCCD, Andor). The HeNe laser source was from Thorlabs (HNL210LB); a 10X microscope objective (10X Olympus Plan Achromat Objective, 0.25 NA, 10.6 mm WD, from Thorlabs, RMS10X) and a 50X objective (50X Objective, 0.55 NA, 13 mm WD, from Newport, MLWD-50X) were separately used during spectra acquisition. Xe light was used for calibration from 100 to 4000 cm^{-1} spectral window before experimental measurements. The detailed description and scheme can be found in our previous work[2].

1.4 DEMS measurements

A mass spectrometer system[3] and an electrochemical dual thin-layer flow cell were used for differential electrochemical mass spectrometry (DEMS) measurements. The details of the system can be found in another work[4]. The Cu (poly) electrode preparation method was used in the same way as described in section 1.2 of SI. The CO_2 reduction in CO_2 saturated 0.1 M $KHCO_3$ solution on a bulk Cu electrode (polished by 85% H_3PO_4 for 1 min.) was studied with DEMS in the dual thin-layer flow cell. The scan rate was 50 mV/s.

2. Extended results, analysis and discussion

2.1 Discussion on the background subtraction of Raman spectra

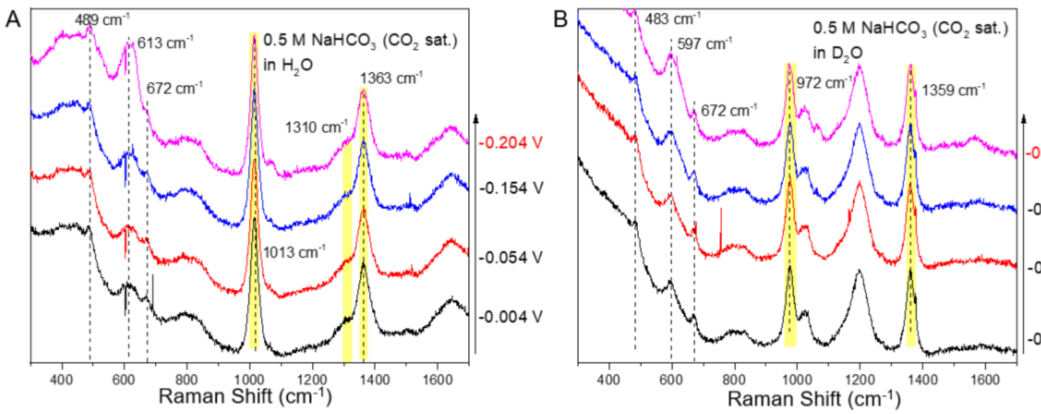

Figure S2. Background Raman spectra selection. (A) Raw (un-subtracted) potential dependent SHINERS spectra in H_2O. (B) Raw (un-subtracted) potential dependent SHINERS spectra in D_2O. The Raman peak of bicarbonates is highlighted in yellow.

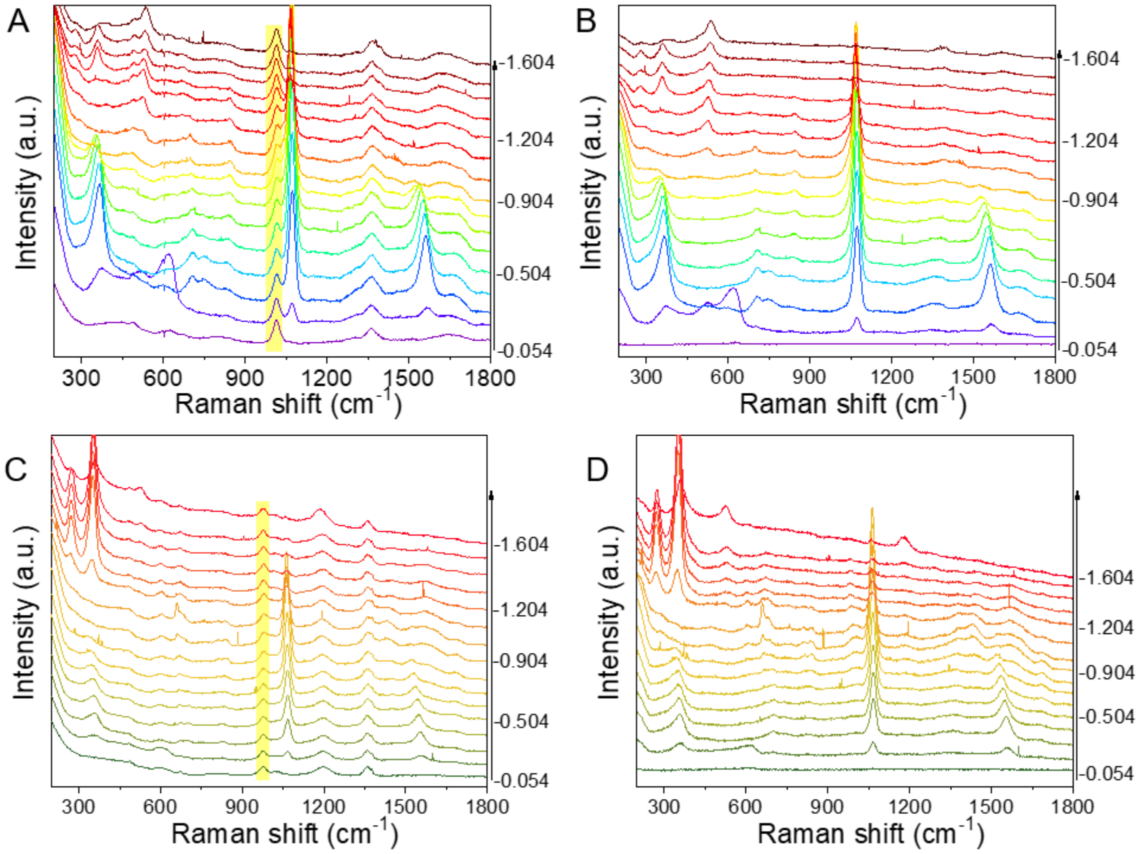

Figure S3. Spectra before and after Background subtraction. Comparison of (A) unsubtracted and (B) subtracted Raman spectra in H_2O; and (C) unsubtracted and (D) subtracted Raman spectra in D_2O. Raman peaks of bicarbonates are highlighted in yellow in A and C.

To facilitate the analysis of potential-dependent spectral features, a background subtraction was conducted. As shown in **Figure S3**, at potentials more positive of -0.204V, the Rama spectra show negligible potential dependence, and as shown in Figure S4, at potentials more negative of -0.204 V, significant potential-dependent features appear. Therefore, the spectrum at -0.004 V is taken as a reference and the raw (un-subtracted) spectra are subtracted by this reference spectrum to remove potential independent spectral components. For example, the main peaks of bicarbonate, ~1020 cm^{-1}, ~1370 cm^{-1} in H_2O and ~980 cm^{-1}, ~1360 cm^{-1} in D_2O, highlighted as yellow color in **Figure S2** and **Figure S3**(A and C), can be nearly completely removed in the subtracted (difference) spectra. This indicates that in our system of 0.5 M $NaHCO_3$ solution, most observed bicarbonate Raman peaks are caused by species in the bulk solution, and there is negligible signal due to adsorbed bicarbonate. The assignment of these peaks agrees well with bicarbonate peaks in bulk solution (**Figure S4**).

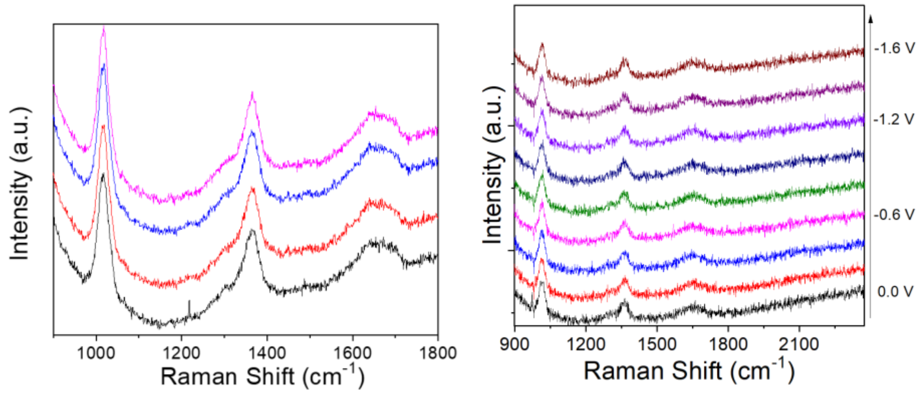

Figure S4. Raman spectra of solution *(bulk)* phase *in* 0.5 M $NaHCO_3$ (CO_2 sat.) *water* solution. Left: different spectra of different colors represent the different sample spots; Right: potential dependent Raman spectra of solution(bulk)-phase.

During spectral background subtraction, we observed a persistent feature near 1020 cm^{-1} in H_2O and 980 cm^{-1} in D_2O at potentials more negative than -1.0~–1.2 V, which could not be fully removed. This signal is attributed to surface-bound bicarbonate species (HCO_3^-), indicating its presence in addition to carbonate (CO_3^{2-}) under strongly reducing conditions.

2.2 Additional DFT calculated Raman spectra of key species

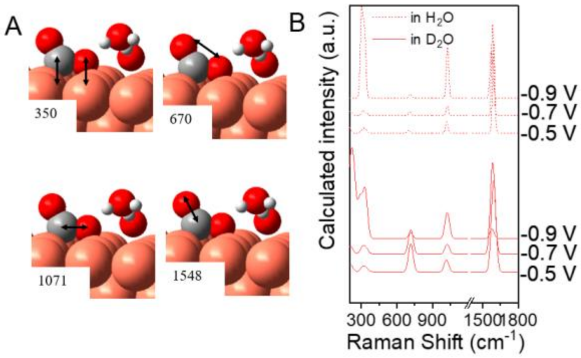

Figure S5. DFT calculation of *COO⁻ with explicit water. (A) DFT calculated the structure of *COO⁻ with explicit water. (B) corresponding calculated Raman spectra of *COO⁻ in H_2O/D_2O.

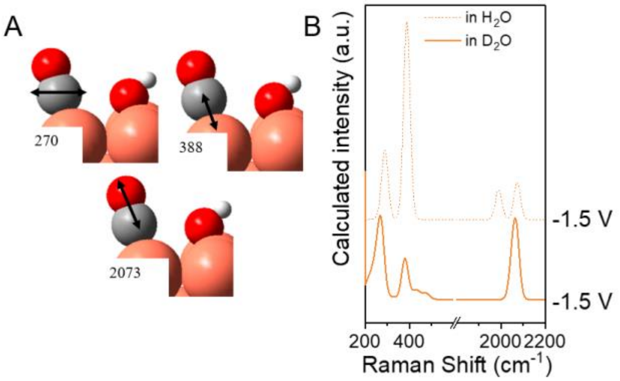

Figure S6. DFT calculation of *CO with nearby *OH. (A) DFT calculated structure of *CO with nearby *OH. (B) corresponding calculated Raman spectra of *CO in H_2O/D_2O.

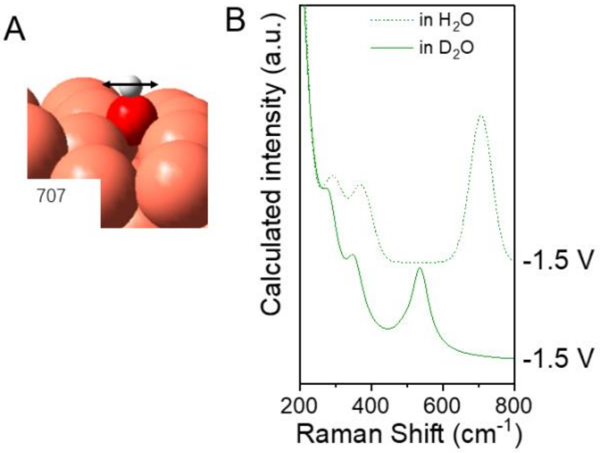

Figure S7. DFT calculation of *OH. (A) DFT calculated the structure of *OH. (B) corresponding calculated Raman spectra of *OH(*OD) in H_2O/D_2O.

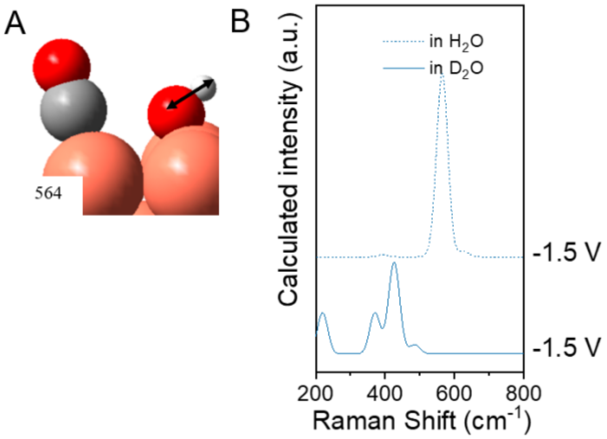

Figure S8. DFT calculation of *OH with nearby *CO. (A) DFT calculated the structure of *OH with nearby *CO. (B) corresponding calculated Raman spectra of *OH(*OD) in H_2O/D_2O.

2.3 Summary of the Raman peaks assignment

The potential dependent frequency shifts of observed species seen in **Table S1** indicate that they are surface adsorbed and can experience electric field changes. **Table S1** also shows the H/D isotope effect of these species. The experimentally observed higher and lower frequency modes of COO^- both show 5-10 cm^{-1} frequency differences in H_2O and D_2O, which indicate this species interacts with protons or water molecules. Similar isotope effects are also observed for adsorbed CO. Therefore, in our DFT models, to account for the adsorbate-water interaction, we include explicit water molecules into the system in addition to an implicit solvent. Zero, two, three, and seven waters were included around COO^- to test how the additional water molecules impacted the spectra. For the higher and lower frequency peaks, small changes of <5 cm^{-1} were seen with the inclusion of hydrogen bonding for any number of explicit water molecules. The lack of notable spectral change beyond maximum hydrogen bonding to the COO^- meant a model with two waters was sufficient.

CO_3^{2-} Peaks. We assign the Raman peak at ~1071 cm^{-1} in the potential range of -0.2 V to -1.3 V to the symmetric stretching mode of bidentate adsorbed $*CO_3^{2-}$(Figure 2B), based on its agreement with the reported Raman spectra of carbonate in the solution phase[5]. This peak shows

a ~5 cm^{-1} frequency shift over the potential range, indicating that the absorbed carbonate is able to sense changes in the electric field of the double layer. Specifically, it shows a red shift from -0.2 to -1.2 V and a blue shift from -1.2 to -1.6 V (**Error! Reference source not found.B**); this potential dependent frequency shift is also corroborated by our DFT calculations (**Figure 2B**). Furthermore, this peak also shows a ~5 cm^{-1} red shift from H_2O to D_2O (**Error! Reference source not found.B**), suggesting that its frequency is affected by interactions with nearby water molecules. This effect has not been reproduced by the DFT calculation, which may be due to insufficient water molecules near the carbonate in the computation model. Our calculation indicates that $*CO_3^{2-}$ can be either bidentate or monodentate when adsorbed on Cu surfaces, consistent with a previous literature report.[6]

Table S1. Summary of measured and computed Raman peaks of key species on Cu(pc)

Species	Vibrational modes	Solvents	Measured Frequency (cm^{-1})	Computed Frequency (cm^{-1})	Measured frequency shift (cm^{-1}/V)
$*CO_3^{2-}$	ν (a1')[34,36]	H_2O	~ 1076 (-0.4 V)	1022 (-0.9 V)	8.0 ± 0.4 (-0.4 to -1.2 V)
		D_2O	~ 1071 (-0.4 V)	1022 (-0.9 V)	8.6 ± 0.3 (-0.4 to -1.2 V)
$*COO^-$ Na^+	ν (Cu-C)	H_2O	~ 360 (-0.4 V)	315 (-0.5 V)	34.8 ± 2.1 (-0.2 to -0.8 V)
		D_2O	~ 350 (-0.4 V)	322 (-0.5 V)	29.7 ± 1.0 (-0.2 to -0.8 V)
$*COO^-$ Na^+	$ν_a$ (C=O)	H_2O	~ 1555 (-0.4 V)	1592 (-0.5 V)	62.6 ± 2.1 (-0.2 to -0.8 V)
		D_2O	~ 1548 (-0.4 V)	1592 (-0.5 V)	64.3 ± 1.4 (-0.2 to -0.8 V)
Cu-OH (non-hydrogen bonded)	δ (Cu-O-H)	H_2O	~ 700 (-0.5 V)	707 (-0.5 V)	Unobtained due to overlapping
		D_2O	~ 670 (-0.5 V)	536 (-0.5 V)	19.7 ± 2.1 (-0.5 to -1.15 V)
Cu-OH in $CuO_x(OH)_y$	ν (Cu-O)	H_2O	~ 527 (-1.1 V)	565 (-0.5 V)	-26.1 ± 0.8 (-1.0 to -1.5 V)
		D_2O	~ 523 (-1.1 V)	485 (-0.5 V)	-7.5 ± 1.6 (-1.0 to -1.5 V)
*CO	ν (Cu-C)	H_2O	~272 (-1.25 V)	289 (-1.5 V)	-8.6 ± 2.8 (-1.25 to -1.6 V)

		D_2O	~280 (-1.25 V)	268 (-1.5 V)	-3.8 ± 0.4 (-1.25 to -1.6 V)
*CO	ρ (Cu-C-O)	H_2O	~358 (-1.25 V)	389 (-1.5 V)	-12.0 ± 4.0 (-1.25 to -1.6 V)
		D_2O	~350 (-1.25 V)	380 (-1.5 V)	-11.3 ± 0.2 (-1.25 to -1.6 V)
*CO	ν (C-O)	H_2O	~2020 to 2100	~2020 to 2100	Multiple modes (Seen in SI 2.4)
		D_2O	~2020 to 2100	~2020 to 2100	

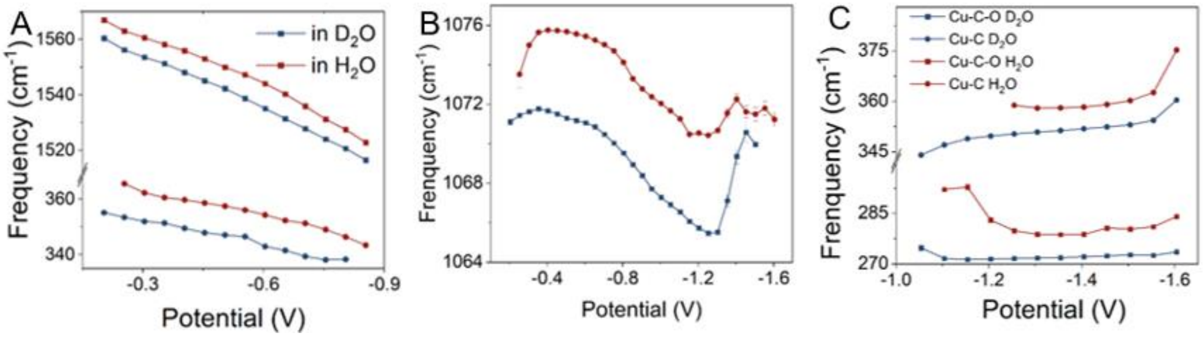

Figure S9. Potential dependent frequency shifts of carbon-containing species in H_2O and D_2O. (A)COO^-(1540 and 360 cm^{-1}), (B)carbonate (1071 cm^{-1}), (C)Cu-CO (280 and 370 cm^{-1}).

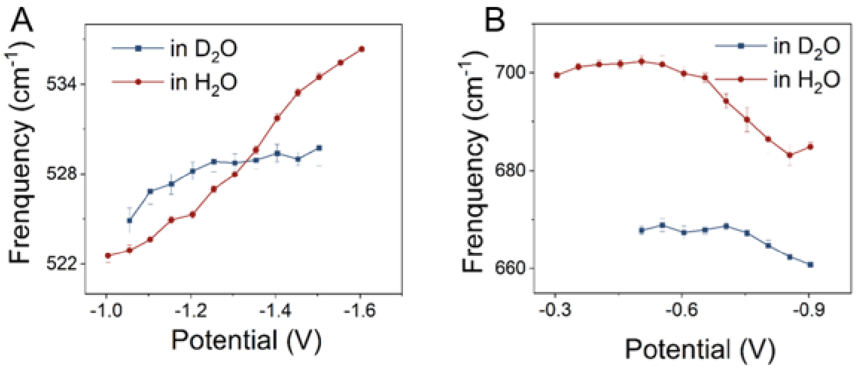

Figure S10. Potential dependent frequency shifts of OH-containing species in H_2O and D_2O. (A) hydrogen bonding Cu-OH(OD) bending (520 cm^{-1}) and (B) free Cu-OH(OD) (670 cm^{-1}).

HCO_3^- Peaks. In the raw Raman spectra before subtraction, shown in **Error! Reference source not found.A** and **C**, we observe an intense peak at ~980 cm^{-1} in D_2O and ~1018 cm^{-1} in H_2O, which can be attributed to the in-plane C-O stretching mode of uncoordinated bicarbonate in the bulk solution (**Figure S4**) [7]. This is consistent with the observation that this peak shows negligible dependence on the applied bias and has negligible intensity in the subtracted spectra shown in **Figure 2B** and **Figure S3.** Spectra before and **after Background subtraction.** As shown in **Figure S4**, in the bulk solution phase, Raman spectra are dominated by bicarbonate (1018 cm^{-1}) with negligible carbonate contribution (1055 cm^{-1}), consistent with the expected concentration ratio ($[H_2CO_3]$ /$[HCO_3^-]$ /$[CO_3^{2-}]$≈2:8:0.01 at pH=7.2[8]). However, our raw Raman spectra at the interface (**Figure S3**) show a much lower HCO_3^-/ CO_3^{2-} intensity ratio compared to the bulk solution, and the subtracted Raman spectra show negligible HCO_3^- intensity. These results suggest that there is a preferential accumulation of CO_3^{2-} on the surface, consistent with literature reports[9–11].

****COO⁻ Peaks.*** Our experimental and computational results suggest that the ~1540 cm^{-1} and the ~360 cm^{-1} peaks should be assigned to $*COO^-M^+$, a cation M^+ stabilized adsorbed $*COO^-$, rather than other literature assignments such as carbonate [12–14], malachite [15], or *COOH [16]. A detailed discussion of the reasons for supporting the $*COO^-M^+$ assignment and ruling out other possibilities can be found in SI 2.4.

****CO Peaks.*** We assign the broad Raman peak at ~2080 cm^{-1} from -0.904 V to -1.604 V to the C-O stretching mode of *CO. This broad Raman band consists of three overlapping *CO species: ~2030 cm^{-1}, ~2060 cm^{-1} and ~2090 cm^{-1}. These species show different potential dependent frequency tuning rates (shown in SI 2.6.), indicating they are *CO adsorbates in different chemical environments. In previous studies, vibrational peaks in the 2000 to 2100 cm^{-1} region are assigned to atop *CO, but multiple reasons have been suggested for the wide distribution of vibrational frequencies[17,18], including a distribution of the coordination number of the Cu sites[18] and the extent of interaction of surrounding *OH group[17]. In our analysis, we fit these CO modes and obtain the total intensity of all atop *CO bands. Our DFT calculations and experiments also identify lower frequency modes at 270 cm^{-1} and 350 cm^{-1} (**Figure 2D**). We assign these to the Cu-C-O bending (Cu-CO restricted rotation) and the Cu-C stretching modes of Cu-CO respectively, consistent with previous literature reports[17–19].

Cu_2O and Cu-OH/OH^- Peaks. The ~623 cm^{-1} peak (brown) appears between -0.054 V and -0.204 V (**Figure 1B**) and shows negligible frequency change in H_2O. This peak is assigned to Cu_2O and its disappearance at more negative potential has been attributed to the reduction of Cu_2O to Cu[20,21]. Immediately following the decline of the ~623 cm^{-1} peak intensity, a rise of $*COO^-$ and $*CO_3^{2-}$ peaks are observed (**Figure 3**). This suggests that the reduction of the electrode surface from Cu_2O to Cu initiates CO_2RR and the adsorption of carbon-containing species.

In addition, the ~660 cm^{-1} peak (green shaded peak in **Figure 1B** and **2A**) is assigned with the bending mode of Cu-O-H(D) that is minimally engaged in hydrogen bonding, and the ~523 cm^{-1} peak (blue shaded peak in **Figure 1B** and **2A**) is assigned with the OH stretching mode of $CuO_x(OH)_y$. The ~660 cm^{-1} peak shows a relatively consistent isotopic H/D effect, while the ~523 cm^{-1} peak position and its H/D shifts can be greatly affected by the environment (e.g. the population of adsorbed CO, **Figure S12-S14**); therefore, we believe the 523 cm^{-1} peak should be a special mode that possibly hydrogen bonds with surface *CO such that its spectral feature has the property of adsorbing *O with diminished H/D isotopic shifts. Further reasoning and discussion for the assignments can be found in SI 2.5.

2.4 Additional discussion on $*COO^-$ peaks assignments

Assignment to $*COO^-M^+$. We assign the Raman peaks at ~1540 cm^{-1} and ~360 cm^{-1} to $*COO^-M^+$, agreeing with a recent SERS study by Chernyshova et al.[22] The ~1540 cm^{-1} peak, which we argue based on frequency and frequency shift matching to DFT calculation is from an asymmetric C=O stretching mode of $*COO^-$. The ~360 cm^{-1} peak, in accordance, should be assigned with the Cu-C stretching mode (**Figure 2B**). The cation M^+ is to stabilize the total structure on the surface. This Raman peak presents itself over a broad potential region (-0.204 to -0.854 V), and since it manifests before the appearance of *CO, it is largely believed to be a possible precursor and the rate-limiting step in the CO_2RR mechanism to make *CO.

We also assign the ~360 cm^{-1} Raman peak to the Cu-C stretching mode of $*COO^-M^+$ with DFT frequency calculations as evidence. In the experiment, this Raman peak exists within the same potential region as the ~1540 cm^{-1} peak, and both the ~1540 cm^{-1} and the ~360 cm^{-1} peaks exhibit a similar change in intensity as a function of potential as well as a similar shift in frequency direction due to Stark effect and possible coverage dependence effects[23] (**Figure 2B**). In detail, in CO_2-saturated 0.5 M $NaDCO_3$, the ~1540 cm^{-1} and the ~360 cm^{-1} peaks both grow in intensity

from -0.15 and -0.5 V and maximize around -0.5 V. In addition, their potential dependent frequency tuning rates are 64.3 ± 1.4 and 29.7 ± 1.0 cm^{-1}/V respectively (**Table S1**). These results suggest that the ~1540 cm^{-1} and the ~360 cm^{-1} peaks belong to the same adsorbed species.

Nevertheless, previous studies have presented inconsistent assignments on the chemical identity attributed to the ~1540 cm^{-1} and ~360 cm^{-1} peaks, with the literature reports assigning them to carbonate [12–14], malachite [15], $*COO^-$ [22,24], and *COOH [16]. The challenge is because the peak's frequency range of 1500-1600 cm^{-1} can belong to the C=O bond stretch in many different species, and both Cu-C or Cu-O modes are in the 300-400 cm^{-1} region.

Ruling out carbonate or bicarbonate. We exclude the possibility of assigning the ~1540 cm^{-1} peak to carbonate or bicarbonate based on the following 3 reasons. Firstly, the ~1540 cm^{-1} peak cannot be a solution phase species, as it was not observed in our bulk spectra(**Figure S4**) or any previous studies on bulk carbonate/bicarbonate[7,25]. Secondly, under CO_2 reduction conditions, its intensity potential dependency doesn't change accordingly with known carbonate/bicarbonate peaks. While it increases in intensity from -0.204 to -0.3 V, plateaus from -0.3 to -0.5 V, and then decreases in intensity until -0.854 V (**Figure 3A**), the intensity of the $*CO_3^{2-}$ band at ~1055 cm^{-1} increases at -0.6 V, and that of the HCO_3^- band at ~1018 cm^{-1} shows no potential-dependence. Thirdly, the 1540 cm^{-1} peak is not observed in our pure carbonate solution binding control in Ar-saturated 0.25 M Na_2CO_3 solution (pH = 11.95), as shown in **Figure** . This result is consistent with that of Chernyshova et al.[22]. It should be noted that on other metals with different ion-affinity, such as Au[26] or Pt[10], the 1500-1600 cm^{-1} assignments can be different because many C=O containing species such as carbonate/bicarbonate do have similar stretching modes in the similar frequency region.

We excluded the assignment of the ~360 cm^{-1} Raman peak to carbonate as reported by another paper[14] because of the inconsistency on the potential dependent intensity compared to the known carbonate peak ~1055 cm^{-1} (**Figure 1B and Figure 2B,C**): from -0.85 V to -1.15 V where the ~1055 cm^{-1} peak shows growing intensity, the ~360 cm^{-1} peak has already decreased to negligible intensity. Thus, the ~360 cm^{-1} Raman peak should not be assigned to carbonate species.

Ruling out *COOH. Moreover, we disagree with assigning the ~1540 cm^{-1} peak to anti-symmetric stretching of *COOH. As shown in **Figure S11B**, our DFT calculations of *COOH give prominent vibrational modes at ~302, ~369, ~523, ~800 cm^{-1} and ~1696 cm^{-1}. Examining first the low frequency modes, the calculation predicts that the peaks ~523 and ~800 cm^{-1} show

similar intensities and Stark tuning rate as the observed peak at ~369 cm^{-1}, but these peaks are not observed in the experimental spectra in **Figure S11A** or **Figure 1**. Moving to the high frequency, the calculated ~1696 cm^{-1} peak shows a small frequency shift towards higher frequency in the cathodic scan, while the experimental ~1540 cm^{-1} peak shows a frequency shift in the opposite direction. Regardless of explicit water environment or the presence of Na^{+}, the peak in the higher than experimental >1600 cm^{-1} frequency region persists. Thus, we excluded the possibility of *COOH assignment.

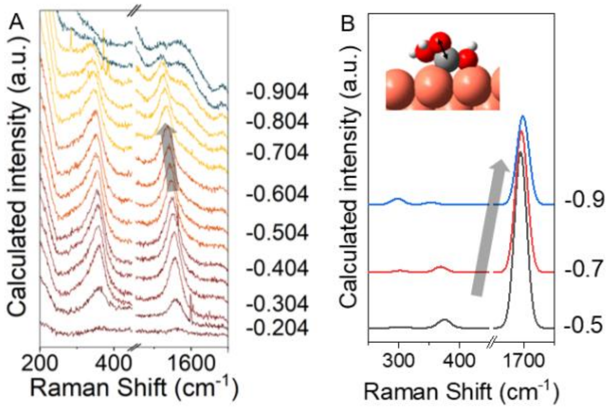

Figure S11. Ruling out the assignment of *COOH. (A) Zoom-in potential dependent spectra at ~1540 cm^{-1} and ~340 cm^{-1} (left, arrow aligned) (B) calculated spectra of *COOH with simulated structure (right, arrow aligned) The different atoms are shown in different colors: C atom grey, O atom red, H atom white. From positive to negative potentials, the experimental results showed a red shift while DFT results showed a blue shift, indicating the mismatched assignment of *COOH.

Ruling out malachite - Peak Presence. The previous work by Jiang et al.[15] reported the assignment of ~ 1070 cm^{-1} and 1540 cm^{-1} to be malachite, a type of Cu hydroxide carbonate species, not the adsorbed carbonate. Other previous work[27,28] has predicted and showed that malachite is an important precursor of Cu surface chemical conversion in altering CO_2 reduction activity. Purposely prepared malachite[28] shows clear differences in vibrational spectral features compared with Cu or Cu_2O and exhibits different product selectivity. Although the malachite Raman spectra show peaks at ~ 1070 cm^{-1} and ~1540 cm^{-1} peaks, as well as at ~360 cm^{-1} and ~700 cm^{-1}, our observed Raman peaks in this region cannot be assigned to malachite based on the following reasons.

We cannot assign the ~1540 cm^{-1} peak to the anti-symmetric stretching of carbonate in malachite because: (1) under CO_2 reduction condition, the potential dependent relative intensity

changes of "malachite-related" vibrational modes are not in sync, i.e. from -0.3 V to -0.8 V, ~1070 cm^{-1} peak stops increasing while ~1540 cm^{-1} and ~360 cm^{-1} continues to grow; (2) the missing ~1540 cm^{-1} peak in malachite-enriched sample control under CO_2 reduction condition[15], even though the purposely malachite-enriched sample shows exactly the same spectra features of malachite, but the potential dependent spectra showed no peaks at ~1540 cm^{-1}; (3) the missing ~1540 cm^{-1} peak in 0.25 M Ar saturated Na_2CO_3 carbonate control (**Figure**) or their carbonate reported control[15].

For the ~360 cm^{-1} peak, we disagree with its possible assignment on O-Cu-O bending modes of malachite[15] due to: (1) the different direction of ~360 cm^{-1} peak potential dependent frequency shift: while a noticeable red shift (from -0.2 V to -0.8 V) of around ~30 cm^{-1}/V is observed for the ~360 cm^{-1} Raman peak, the O-Cu-O bending mode of malachite bulk species does not see any frequency shifts; (2) in our H/D labelling experiment of CO_2-saturated 0.5 M $NaH(D)CO_3$, the ~360 cm^{-1} peak in H_2O exhibits an isotope shift to ~350 cm^{-1} in D_2O, indicating stronger interaction of surface species and explicit solvents. This 10 cm^{-1} isotope shift disagrees with a previous report that suggests that bulk malachite system does not have a significant H/D shift[15].

With the listed reasons, we support the assignments of surface carbonate (~1070 cm^{-1}) and $*COO^-$ (~360 cm^{-1} ~1540 cm^{-1}) rather than malachite.

Ruling out malachite – Potential Dependent Intensity Based Mechanism. In the main text Figure 3 and SI **Figure S16**, we plot the potential dependent intensity changes of different species and attribute them to carbonate competitive binding mechanism. For the sake of completeness, in this section, we discuss related observations by other previous reports[15,29] , and why we believe these observations have been misattributed to malachite.

Malachite-precursor mechanism. In a literature mechanism[15], a similar potential dependent intensity change to what is seen in our measurements was associated with the loss of the 1540 cm^{-1} peak which was assigned to malachite, and the growth of a *CO peak. Testing this, we see similar results for 0.1 M $NaHCO_3$ (CO_2 saturated, pH=6.7)[22] or 0.1 M $KHCO_3$ (CO_2 saturated, pH=6.8)[15], where the interchange of disappearing of 1540 cm^{-1} peak and onset of *CO can overlap from 0.1 V to -0.1 V (vs. RHE); however, our measurement of 0.5 M $NaHCO_3$ (CO_2 saturated, pH=7.2), has a later *CO onset at around -0.2 V to -0.25 V vs. RHE, where the peak of

~1540 cm^{-1} starts to decreases at ~ 0.1 V and is no longer observed at -0.1 V. This leads to a gap between the supposed malachite disappearance at 1540 cm^{-1} and *CO appearance in our measurements (**Figure S16** or **Figure 3A**), without any other products appearing during the range, further supporting our assignment of this 1540 cm^{-1} peak to $*COO^{-}$ over malachite.

Malachite-formation induced local pH change mechanism. Another interpretation of the potential dependent carbonate Raman peak intensity change is reported by Henckel et al.[29] . In their work, the potential dependent carbonate peak intensity change was similarly observed but attributed to surface pH change. This led them to then utilize the intensity ratio between carbonate and bicarbonate as a measure of pH which would further be used to justify a mechanism for malachite formation. We believe this train of logic is flawed. While we agree the surface pH definitely changes during cathodic potential sweeps, and certainly this leads to Cu oxide reduction and other side reactions that must occur to change the local pH; we disagree with attributing a significant amount of the potential-dependent intensity change of carbonate to the pH change based on three reasons. First, a well accepted SEIRAS study[30] on a Au surface, demonstrated the vibrational modes for carbonate and bicarbonate at ~1363 to ~1400 cm^{-1} could be utilized to quantify the surface pH changes, as the bicarbonate and carbonate were not competitive with adsorbed *CO [31] on a Au surface. However, it has also been proven that[31,32] carbonate on Cu shows specific adsorption and greatly affects other adsorbates, therefore meaning increased intensity of carbonate cannot be seen as solely resulting from pH change. Second, we observed at least 8-9 cm^{-1}/V Stark shifts for carbonate in each of our control experiments, giving strong evidence of adsorbate nature that would feel and respond to an electric field change, suggesting our experiments align with the previous Cu studies not the Au. Third, as **Figure 24** shows, our Ar saturated 0.25 M Na_2CO_3 control with pH = 11.95 showed a similar potential-dependent intensity change for carbonate as seen under 6-7 pH. This is unlikely to occur if carbonate could act as a pH probe. Therefore, with these three reasons, we believe the carbonate in our system exhibits more features of adsorbates rather than a local pH probe, which may not therefore act as a reliable determiner of whether the surface has the correct pH for malachite formation. Still, we would like to see more studies similar to the gradient vibrational detection study[11] to disentangle the contribution from the gradient pH change and that of adsorption.

2.5 Additional discussion on Cu-OH related species assignments

We assign the ~660 cm^{-1} peak (green) to be the bending mode of Cu-O-H(D) that is minimally engaged in hydrogen bonding, which is also consistent with the assignments by Bodappa et al.[21] In our experiments, we find the ~660 cm^{-1} peak in D_2O shifts to around 700 cm^{-1} in H_2O. However, in H_2O, the precise peak analysis is difficult as this ~700 cm^{-1} peak may overlap with other modes associated with carbonate or *COO^- [22,33]. Our DFT calculations find that Cu-O-H bending mode with hydrogen bonding (with H_2O or CO) exhibits a 678 or 568 cm^{-1} peak that shifts to ~707 cm^{-1} for that without hydrogen bonding. Thus, we tend to assign it with the Cu-O-H(D) bending mode of a minimally hydrogen-bonding engaged Cu-OH(D). We assign the ~523 cm^{-1} (blue) peak to be the bending mode of a Cu-O-H in a OH against an O containing species, $CuO_x(OH)_y$, due to its smaller isotope shift and more negative onset potential when more CO is added to the system.

In Ar-saturated 0.01 M KOH/KOD solution (**Figure S12**), the blue peak and green peak have similar onset potentials. In addition, the blue peak exhibits an H/D isotopic shift as large as 30 cm^{-1} (~520 cm^{-1} with H, ~490 cm^{-1} with D), which is consistent with the previous report of Cu-OH species in Cu electrooxidation system. In calculations this H/D shift is overexaggerated, likely due to lack of explicit solvent. In comparison, in CO_2-saturated 0.5 M $NaHCO_3$ solution, the blue peak appears at a more negative potential than the green peak (-1.154 V vs. -0.704 V for the green peak), and it shows a significantly smaller 6-7 cm^{-1} isotopic shift (~527 cm^{-1} with H, ~523 cm^{-1} with D, **Figure S13**). In comparison, in CO_2-saturated 0.5 M $NaHCO_3$ solution, the blue peak appears at a more negative potential than the green peak (-1.154 V vs. -0.704 V for the green peak), and it shows a significantly smaller 6-7 cm^{-1} isotopic shift (~527 cm^{-1} with H, ~523 cm^{-1} with D, **Figure S13**). When even more CO is in the system, in CO-saturated 0.01 M KOH solution, the blue peak has an even smaller isotopic shift of 2-3 cm^{-1} (~532 cm^{-1} with H, ~530 cm^{-1} with D, **Figure S14**).

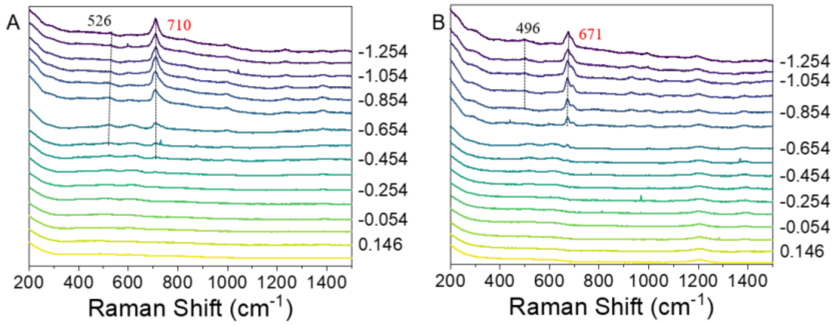

Figure S12. *H/D Raman spectra of OH without CO.*(A) Raman spectra in Ar saturated 0.01 M KOH. (B) Raman spectra in Ar saturated 0.01 M KOD, O-H stretching mode is labelled by the black, Cu-O-H bending mode is labelled by the red color. O-H stretching presents 526 cm^{-1} in H_2O, 496 cm^{-1} in D_2O at -1.254 V, Cu-O-H bending presents 710 cm^{-1} in H_2O, 671 cm^{-1} in D_2O at -1.254 V.

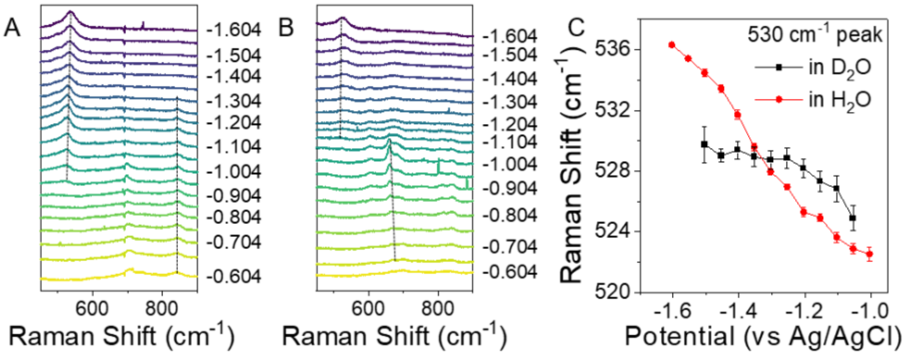

Figure S13. H/D Raman spectra of OH with medium level of CO. (A) in CO_2 saturated 0.5 M $NaHCO_3$. (B) in CO_2 saturated 0.5 M $NaDCO_3$. (C) Comparison of the potential dependent frequencies of the O-H stretching mode in H/D solutions.

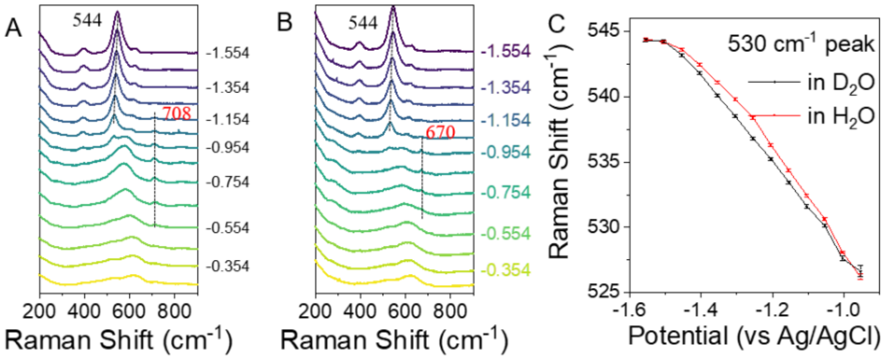

Figure S14. H/D Raman spectra of OH with high level of CO. (A) in CO saturated 0.01 M KOH. (B) in CO saturated 0.01 M KOD. (C) Comparison of the potential dependent frequencies of the O-H stretching mode in H/D solutions.

Given the correlation between the green and blue peaks in the CO-purged system, we believe the blue peak in this environment behaves more like a Cu-OH group engaged in hydrogen bonding. When more CO is added to the system, the blue peak behaves more like a Cu-O group, due to its smaller isotope effect. One explanation for this is that the introduction of *CO results in more *CO - *OH hydrogen bonding/interation[17,34], and disrupts the hydrogen-bonding network among *OH and explicit waters or other *OH near the electrochemical interface such that there is less hydrogen coordination to affect Cu-O stretching from Cu-OH. Another explanation is that *CO reduction involves the deprotonation of Cu-OH to form another product, such as Cu-O_{ad} species reported on Cu(111) surface[21]. Hence, we argue that this blue peak belongs more to a $CuO_x(OH)_y$ species.

This assignment aligns with those found in other studies[35,36]. In the scope of our CO_2RR experiments, the appearance of the green peak between -0.504 V and -1.154 V and then the subsequent appearance of the blue peak between -1.104 V and -1.604 V suggests that the electrode surface is changing from Cu_2O to Cu-OH to $CuO_x(OH)_y$. Moreover, given how the rise of the blue peak coincides with *CO onset and $*CO_3^{2-}$ desorption, we propose that these changes in the surface are correlated with CO_2 reduction processes.

2.6 Supplement electrochemical Raman spectra on *CO

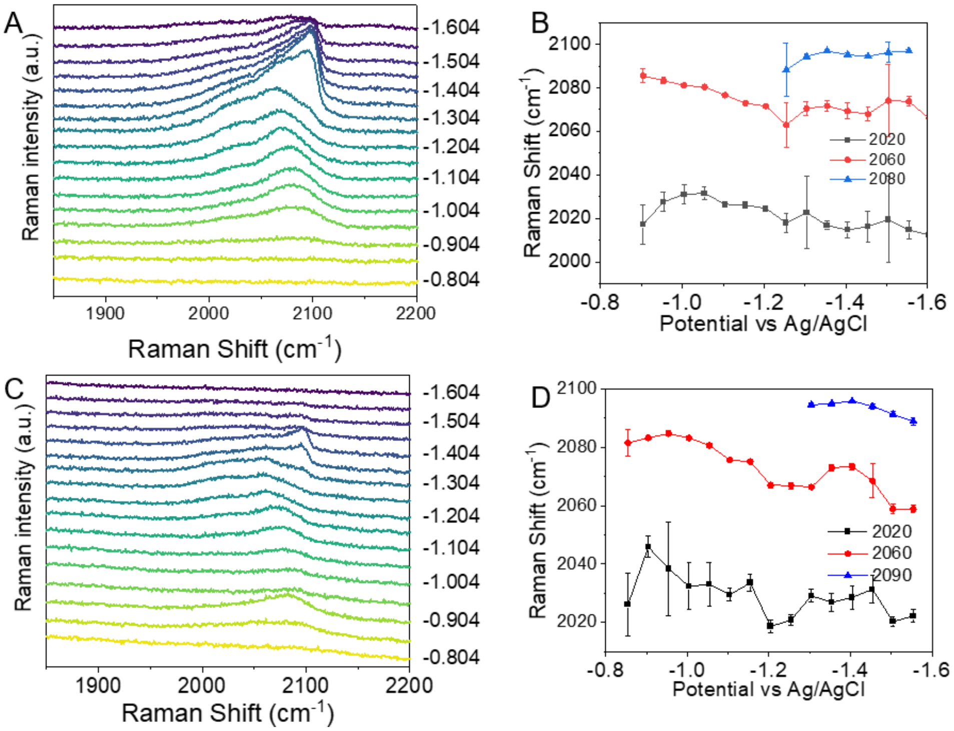

Figure S15. Raman spectra of CO_2 reduced *CO on Cu. (A) potential dependent Raman spectra in 0.5 M $NaDCO_3$ D_2O (CO_2 saturated). (B) Fitted Raman peaks of 3 modes at different frequencies of *CO in 0.5 M $NaDCO_3$ D_2O (CO_2 saturated). (C) potential dependent Raman spectra in 0.5 M $NaHCO_3$ H_2O (CO_2 saturated). (D) The tuning rates of the three modes are difficult to identify as the peak width and frequency both shows potential dependency.

2.7 Additional plot of potential dependent intensity

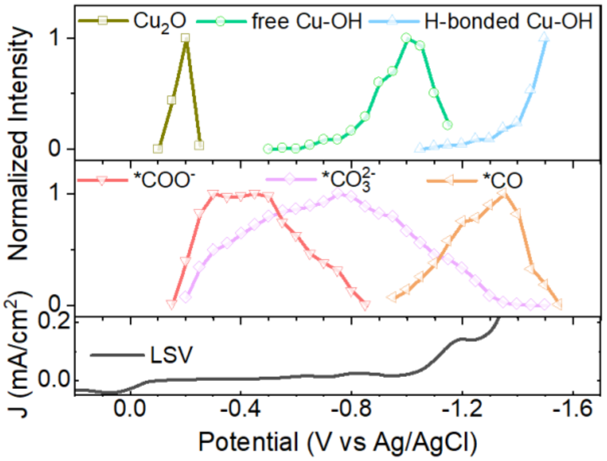

Figure S16. Potential-dependent Raman peak intensity of all Cu-O (top panel) and Cu-C (middle panel) containing species in D_2O. Raman frequency of key species: 623 cm^{-1} (Cu_2O), 520 cm^{-1}

(hydrogen bonding Cu-OH bending) and (B) 670 cm^{-1} (free Cu-OH), 1540 cm^{-1} (*COO^{-}), 1070 cm^{-1} (*CO_3^{2-}), ~2000 to 2100 cm^{-1} (*CO).

2.8 Additional results on *CO_3^{2-} population dependence

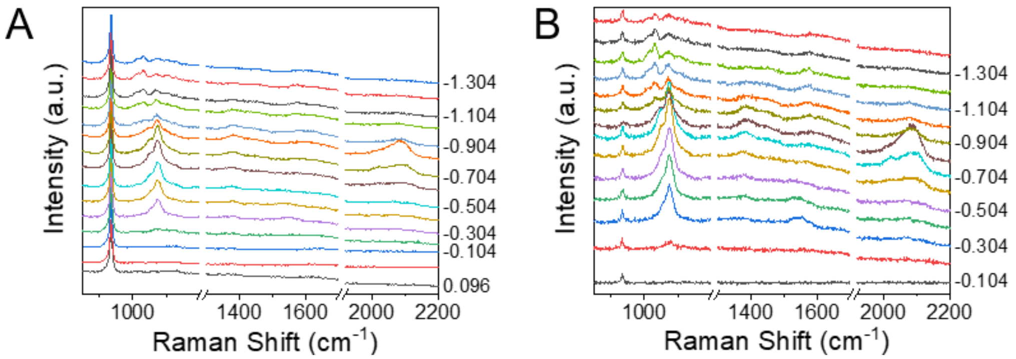

Figure S17. *CO onset potentials using electrolytes with different carbonate concentrations. Comparison of (A) unsubtracted and (B) subtracted Raman spectra (background at -0.004 V) of intermediate carbonate concentration in 0.1 M $NaClO_4$ + 0.4 M $NaHCO_3$ (CO_2 sat. pH=6.8) solution.

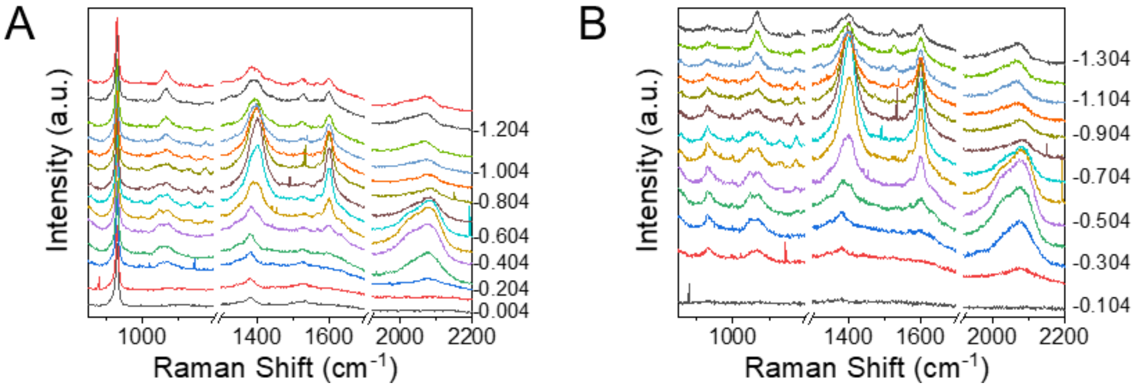

Figure S18. *CO onset potentials using electrolytes with different carbonate concentrations. Comparison of (A) unsubtracted and (B) subtracted **(**background at -0.004 V**)** Raman spectra of low carbonate concentration in 0.5 M $NaClO_4$ (CO_2 sat. pH=4.6) solution.

Notably, in **Figure S18**, the peaks at 1389 cm^{-1} and 1601 cm^{-1} should be related to the *CO reduction to further products, those modes can be correlated with intermediates for *COH or other C_2+ species, such as HOCCOH at 1397 cm^{-1} OCCOH at 1576 cm^{-1}, based on previous literature[37–39].

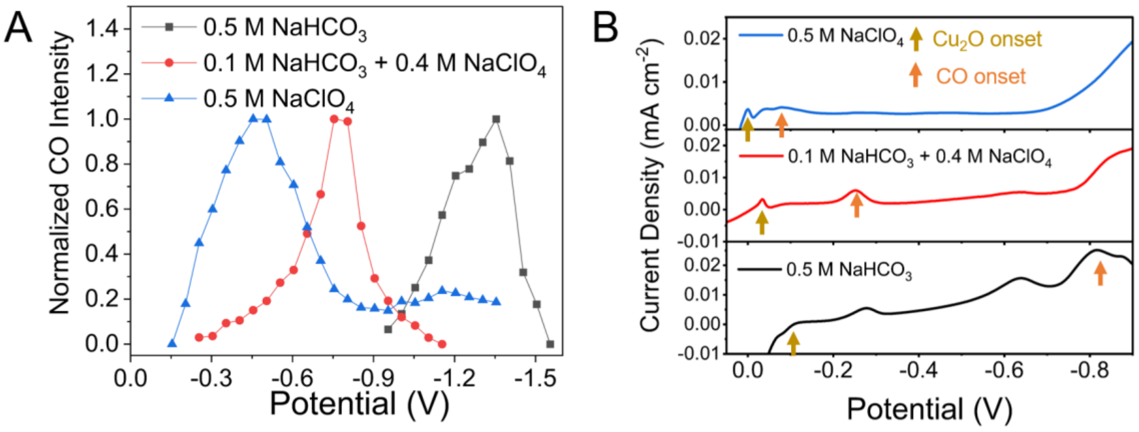

Figure S19. Comparison of CO onset under different amounts of carbonate. (A) Potential dependent *CO intensity in different solutions: 0.5 M $NaHCO_3$ (CO_2 sat. pH=7.2) in black; 0.1 M $NaClO_4$ + 0.4 M $NaHCO_3$ (CO_2 sat. pH=6.8) in red; 0.5 M $NaClO_4$ (CO_2 sat. pH=4.6) in blue. (B) Comparison of linear sweep voltammograms (scan rate 0.4 mV/s) of Cu (poly) in 0.5 M $NaHCO_3$ (CO_2 sat. pH=7.2) in black; 0.1 M $NaClO_4$ + 0.4 M $NaHCO_3$ (CO_2 sat. pH=6.8) in red; 0.5 M $NaClO_4$ (CO_2 sat. pH=4.6) in blue. The onset potentials of Cu2O reduction are marked as dark yellow arrows, and those of CO production are marked as orange arrows.

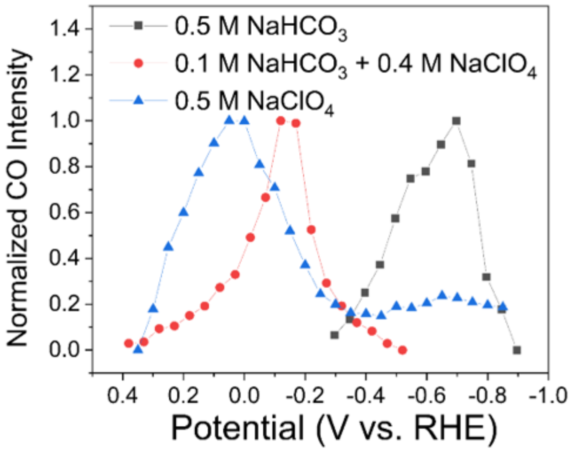

Figure S20. Comparison of normalized intensity of CO in different solutions under RHE scale. 0.5 M $NaHCO_3$ (CO_2 sat. Ph=7.2) in black; 0.1 M $NaClO_4$ + 0.4 M $NaHCO_3$ (CO_2 sat. Ph=6.8) in red; 0.5 M $NaClO_4$ (CO_2 sat. Ph=4.6) in blue.

2.9 Estimation of potentials of zero charge for systems

In our competitive binding model, we think that the strong preferential binding of carbonate on Cu is also controlled by the potential of zero charge (PZC) of the system, to verify this hypothesis, we conducted the potential dependent capacitance measurements of different systems, the PZC can be found at the minimum of the capacitance. The potential dependent differential capacitance was obtained by the potential dependent impedance measurements and followed by the previous report[40]. However, this method can only provide an estimation of the PZC due to the complexity of the system (involve multiple Faradaic reactions), the exact determination may require other methods[28].

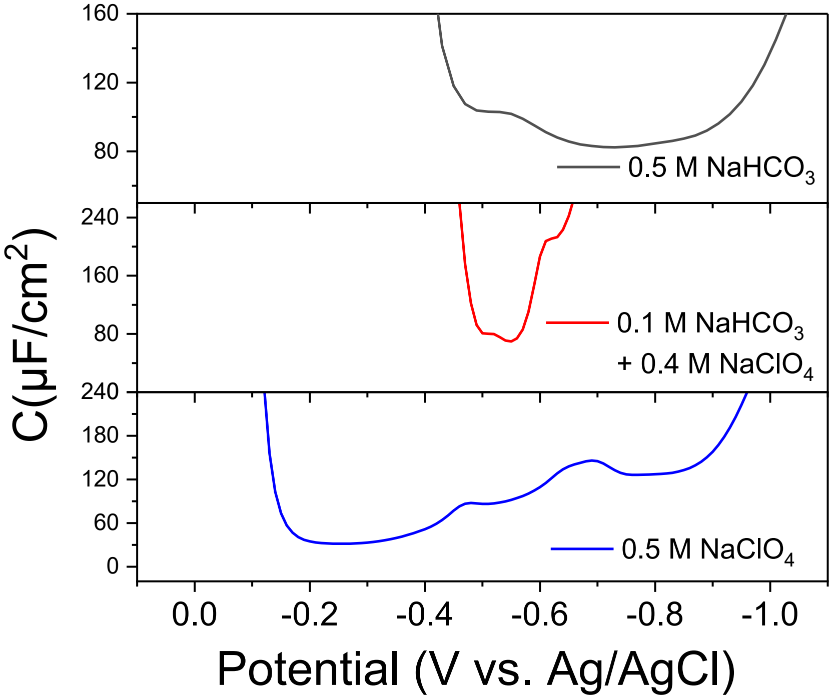

Figure S 21. The differential capacitance for Cu(pc) in different solutions.
Potential dependent capacitance at 25 Hz in different solutions: 0.5 M $NaHCO_3$ sat. with CO_2 (pH=7.2, black line); 0.1 M $NaHCO_3$ + 0.4 M $NaClO_4$ sat. with CO_2 (pH=6.7, red line); 0.5 M $NaClO_4$ sat. with CO_2 (pH=4.6, blue line).

By estimation, the PZC for Cu(pc) in 0.5 M $NaHCO_3$ sat. with CO_2 is roughly around -0.7 to -0.75 V vs. Ag/AgCl, in 0.1 M $NaHCO_3$ + 0.4 M $NaClO_4$ sat. with CO_2 is around -0.55 to -0.60

V, in 0.5 M $NaClO_4$ sat. with CO_2 is around -0.2 to -0.25 V. We observed a pH/carbonate population dependent PZC change, as the carbonate population or the pH becomes smaller, the PZC also decreases. And in each case, the PZC values are also roughly in sync with the potentials for carbonate starting to decrease intensity, thus we believe this carbonate and *CO competitive binding should be directly related with the PZC change of the system. Moreover, the pH dependent PZC change was also reported by previous work in alkaline solutions[28] (from -0.51 V vs. Ag/AgCl at pH=13 to -0.25 V at pH=10 in $NaClO_4$ solutions). In our case, we observed how PZC changes from medium pH (pH=7.2) with more carbonate binding to relatively acidic condition (pH=4.6) and less carbonate binding, thus the variation among our reported values and others' should be mainly induced by different amount of the adsorbates (*carbonate and reaction intermediates) binding.

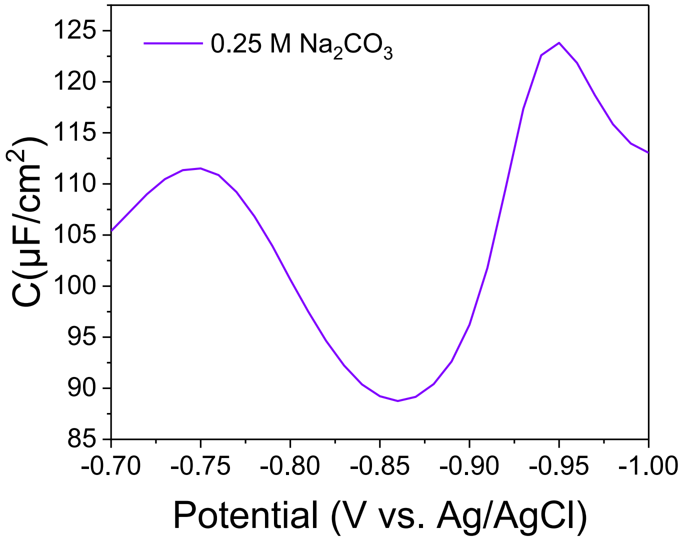

Figure S 22. The differential capacitance for Cu(pc) at 25 Hz in 0.25 M Na_2CO_3 aqueous solution saturated with Ar, pH=11.95.

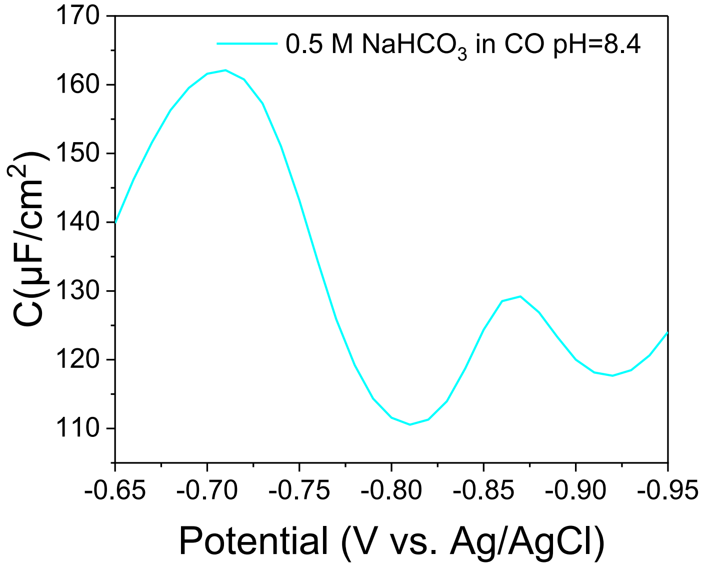

Figure S 23. The differential capacitance for Cu(pc) at 25 Hz in 0.5 M $NaHCO_3$ aqueous solution saturated with CO, pH=8.44.

In a general case where we only consider the adsorption of H^+ or OH^-, the pH effect on the shift of the PZC can be well predicted and studied[41], but the involvement of the other pH-sensitive adsorbates such as carbonate, phosphate, etc., can lead to a unclear complicated case requiring more insights. As the potential dependent intensity change of carbonate is another possible indicator of the PZC shift, we summarize these observations in the following table.

Table S2. Summary of the PZC results.

**Conditions**	**pH**	**Carbonate Peak potential / V vs. Ag/AgCl**	**Electrochemical measured PZC /V vs. Ag/AgCl**	**PZC reported by others in similar conditions**
0.5 M NaHCO3, saturated with CO_2	7.2	-0.75	-0.70 to -0.75	
0.1 M NaHCO3 + 0.4 M NaClO4, saturated with CO_2	6.8	-0.65	-0.55 to -0.60	
0.5 M $NaClO_4$,	4.6	-0.25	-0.20 to -0.25	

saturated with CO_2,				
0.25 M Na_2CO_3,<br>saturated with Ar	11.9	-0.86	-0.85 to -0.90	-0.875 V vs Ag/AgCl Cu(111) in 0.5 M $NaClO_4$ pH=12[42]
0.5 M NaHCO3,<br>saturated with CO	8.4	-0.83	-0.8 to -0.85	

2.10 DEMS results analysis

The DEMS results (seen in **Figure**) showed that the CO_2 reduction on Cu generated large amounts of methane and ethylene, and also small amounts of ethanol as solution volatile products. Formate/formic acid was also formed during CO_2 reduction; however, it is not generally volatile enough to be detected. The amount of CO formed in solution was too small to be detected with DEMS. It should be noted that the decrease of mass spectrometric signal of CO at m/z = 28 at potentials below -1.6 V was due to the decrease in CO_2 concentration near the Cu surface (**Figure S22-Ie**) rather than the decrease in CO concentration, since CO_2 also had a fragment of CO^+. The formation of adsorbed CO (*CO) onset at around -0.20 to -0.25 V vs. RHE, as observed by SHINERS, while most product analysis measurements[43,44] found the onset of bulk CO at a more negative potential than -0.2 V vs. RHE. We think the inconsistence among the bulk and surface measurements may result from: 1) the sensitivity in measuring bulk CO with DEMS in our measurement was not ideal; (2) when the lower overpotentials were applied, there was one adlayer of *CO resulting in an extra energy barrier for continuous CO formation; 3) there is only limited amount of CO at low overpotentials, with no efficient mass transport of *CO to the bulk phase; 4) before it reaches the more negative potential for CO desorption, *CO takes part into *CO reduction, so only sub-monolayer of *CO can be seen. We find some evidence for the fourth reason with

peaks of possible CO reduction intermediates (1178 cm^{-1} OCCO, 1401 cm^{-1} OCCOH, and 1599 cm^{-1} HOCCOH [38,39]) observed in from -0.3 to -1.0 V vs. Ag/AgCl (

Figure S18). It is also evidenced by the DEMS data that methane, ethylene and ethanol were formed as soluble products. And to test other possibilities, a more sensitive fast-stirring spectroscopic study with more sensitive product analysis method may be more helpful in answering this question.

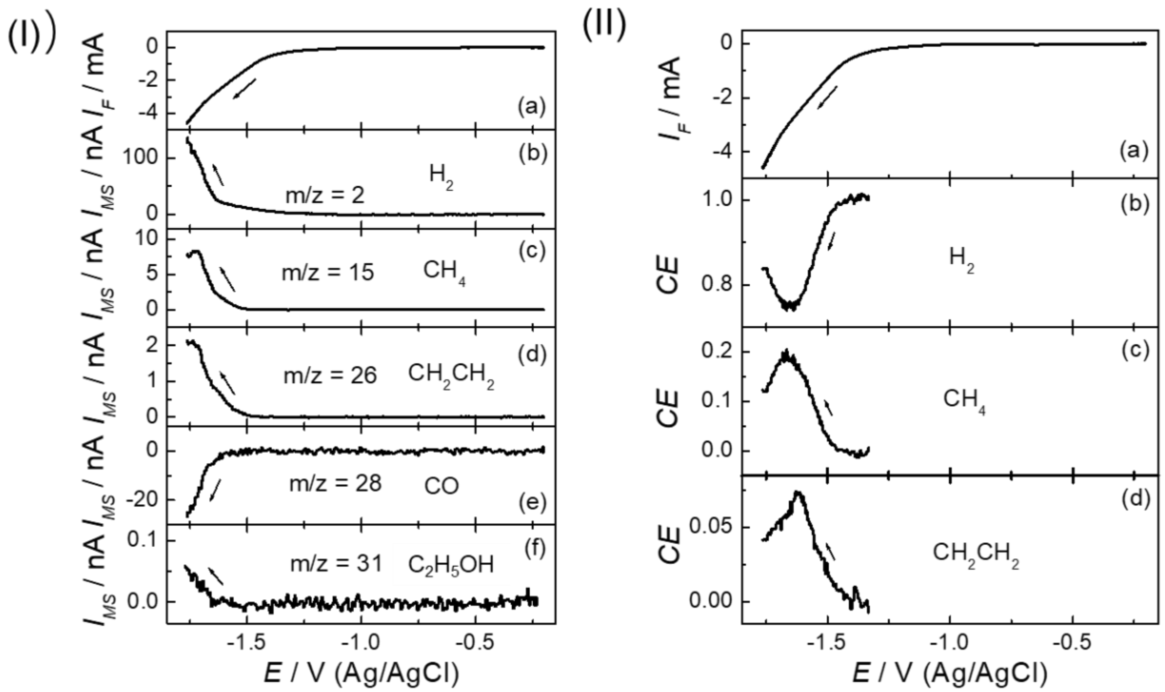

Figure S24. DEMS results analysis. (I) Linear scan voltametric (LSV) profile for CO_2 reduction on a bulk Cu electrode in CO_2 saturated 0.1 M $KHCO_3$ (a) and corresponding mass spectrometric currents at m/z = 2 (b), m/z = 15 (c), m/z = 26 (d), m/z = 28 (e) and m/z = 31(f). Scan rate: 50 mV/s. (II) LSV profile (a) and current efficiencies of H_2 (b), CH_4 (c) and CH_2CH_2 (d) plotted vs. potential.

2.11 Additional results on competitive binding control

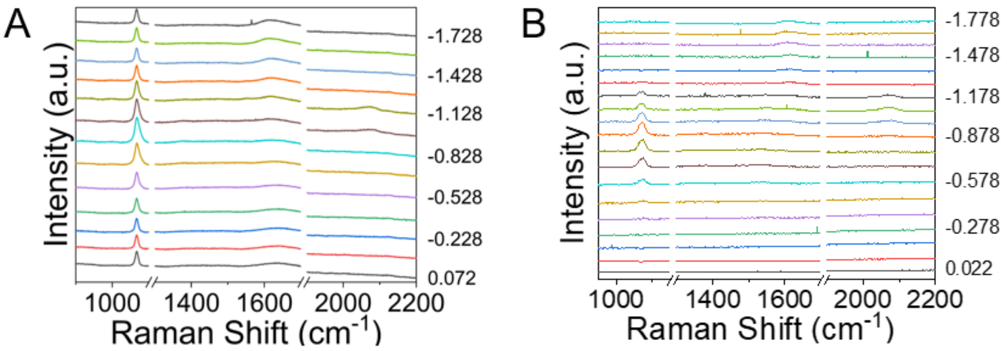

Figure S25. Raman spectra of potential dependent coverage of $*CO_3^{2-}$ in the absence of *CO. (A) Unsubtracted and (B) subtracted Raman spectra Raman spectra in 0.25 M Na_2CO_3 sat. with Ar (pH=11.95).

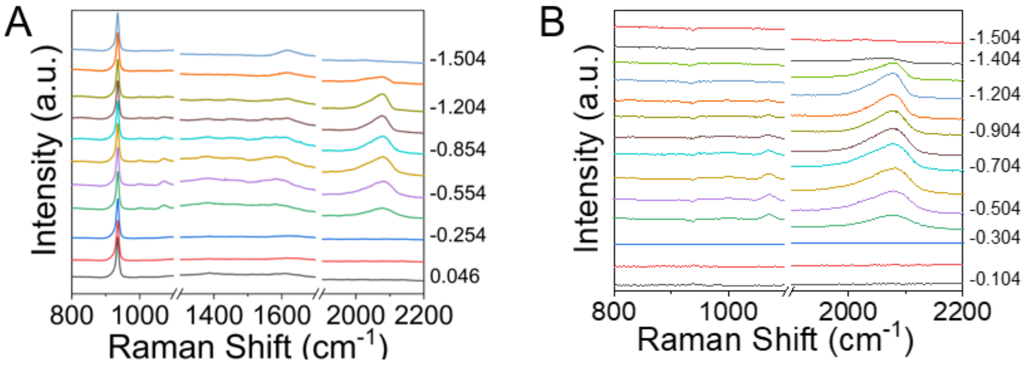

Figure S26. Raman spectra of potential dependent coverage of *CO in the absence $*CO_3^{2-}$. (A) Unsubtracted and (B) subtracted Raman spectra in 0.5 M $NaClO_4$ sat. with CO (pH=7.62).

In the **Figure S26**, there are also some small peaks at 1389 cm^{-1} and 1601 cm^{-1} from -0.554 V to -1.204 V, similarly to the above cases in **Figure S18**, they should be related to the CO reduction products[37–39] (HOCCOH at 1397 cm^{-1} OCCOH at 1576 cm^{-1}).

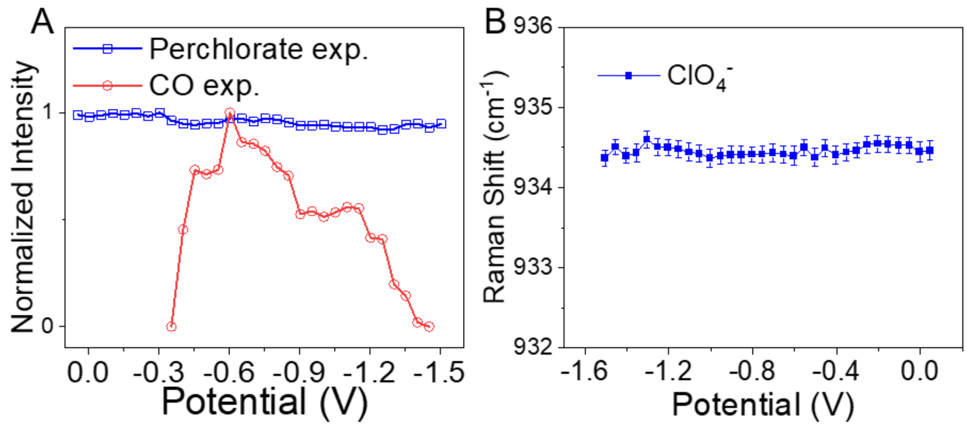

Figure S27. Analysis of the Raman spectra in 0.5 M $NaClO_4$ sat. with CO (pH=7.62). (A) potential dependent intensity of perchlorate and CO intensity. (B) potential dependent frequency of perchlorate anions.

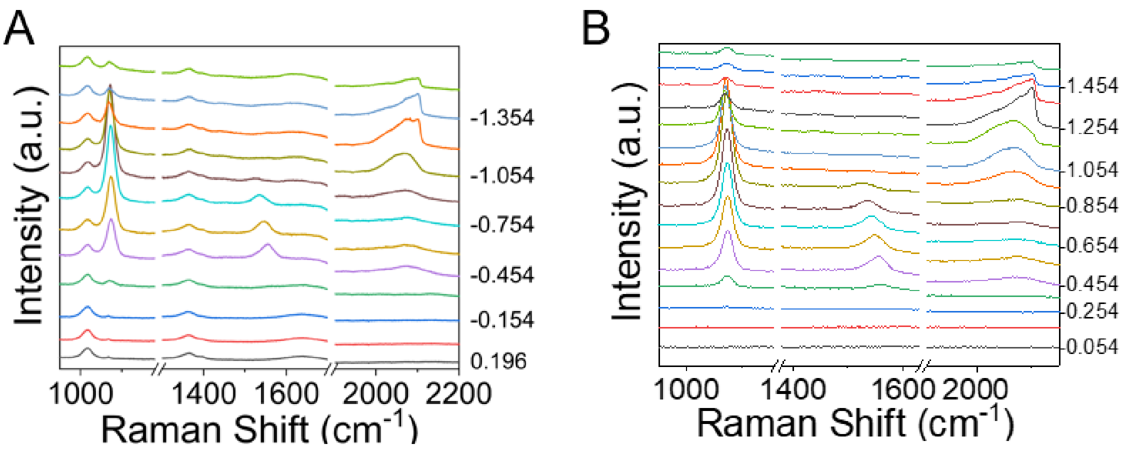

Figure S28. Raman spectra of potential dependent coverage of $*CO_3^{2-}$ and *CO. (A) Unsubtracted and (B) subtracted Raman spectra Raman spectra in 0.5 M $NaHCO_3$ sat. with CO (pH=8.44).

The appearance of $*COO^-$ in the CO-saturated $NaHCO_3$ solution (Figure S28), can be explained by that the existence of CO_2(aq) is necessary for the formation of $*COO^-$. In $NaHCO_3$ solutions, CO_2(aq) is present in equilibrium via the hydrolysis reaction: $HCO_3^- + H_2O \rightleftharpoons H_2CO_3 \rightleftharpoons CO_2(aq) + H_2O$. When some fraction of CO_2(aq) produced through this equilibrium, they can adsorb on the Cu surface and form $*COO^-$ intermediates at certain potentials.

2.12 Discussion on Cu_2O reduction induced carbonate intensity change

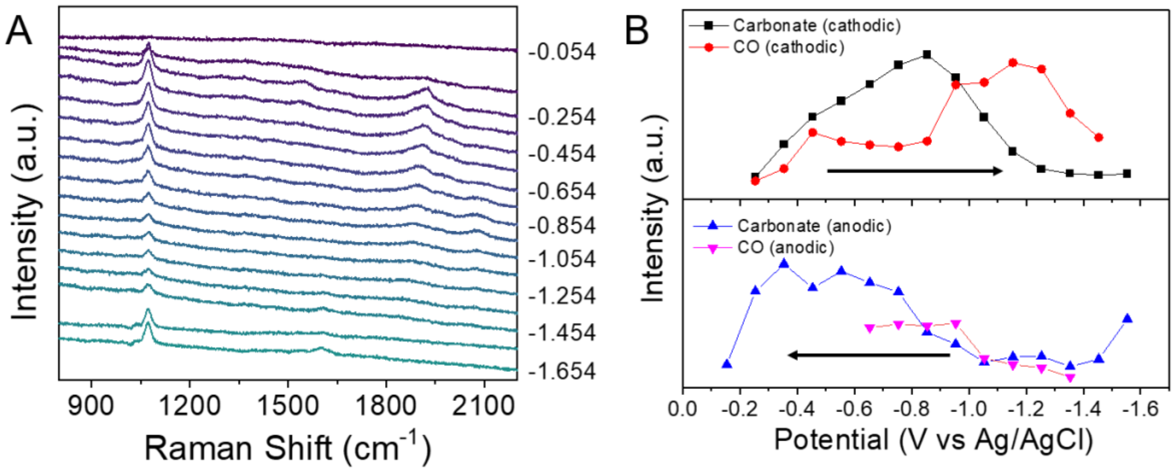

Figure S29. Anodic/cathodic potential dependent coverage of $*CO_3^{2-}$ and *CO. (A) Raman spectra in CO saturated 0.5 M $NaHCO_3$ during an anodic scan. (B) comparison of analyzed intensity of carbonate and CO. The cathodic scan intensity is obtained from **Figure S23**.

The potential dependent carbonate intensity changes in an anodic scan shown in **Figure S26** differs from that in a cathodic scan: while the former has a rapid change from -0.3 V to -0.1 V, the later one shows a gradual increase from -0.1 V to -0.6 V. We believe that the main reason is that the Cu_2O reduction process is slower than the scan rates, while the reverse process of Cu to Cu_2O oxidation is faster.

2.13 Additional MC simulation results

The competitive adsorption on Cu(100) between relevant CO_2 reduction species is modeled with Monte Carlo (MC) simulations. In this model, the change in energy for species *i* to adsorb to a copper site is represented as:

$$\Delta E_i = \Delta E_{PZC,i} + \alpha_i q_i(\varphi + 0.9)$$

where the first term ($\Delta E_{PZC,i}$) is the intrinsic binding energy of adsorbate *i* to Cu at the potential of zero charge (PZC). The second term is the Coulombic interaction of charge (q_i) of adsorbate *i* with the electrode surface of electrical potential (φ). The potential energy is shifted to be zero at the experimentally determined potential of zero charge (around -0.9 V vs. Ag/AgCl in **Figure S *21***). The second term reflects the work to bring a charged adsorbate from the electrolyte bulk to the electrode surface. A constant, α_i, scales this work and may be used to account for other potential-dependent interactions and heterogeneity. The Metropolis-Hastings Algorithm is used to sample the surface ensemble, where the acceptance ratio for the change *A + B → *B + A is:

$$p_{A\to B} = \frac{m_B}{m_A} e^{\frac{-(\Delta E_B - \Delta E_A)}{k_b T}}$$

with m_i being the molality of *i* in the bulk, k_b being the Boltzmann constant, and T is the temperature. Lattice size for a simulation is 200 × 200 sites, and simulations were run for 2×10^6 steps with analysis done on the last 10 percent of simulation.

The experimentally observed V shape potential dependent bicarbonate surface coverage is one remarkable and consistent feature of electrocatalytic studies examining Cu in contact with a bicarbonate electrolyte. This feature persists across all three experiments despite differences in pH and adsorbates (**Figure S30 ai, bi, ci**). The behavior at potential more negative of PZC (~ 0.9V) can be rationalized by the increase Coulomb repulsion between negatively charged surface and carbonate. However, the behavior at more positive potential is puzzling. Within the limitations of our linear model for binding energy, one would expect carbonate to become more attracted to the surface as the electrode is made more positive. In this scheme, carbonate should reach its maximum

surface coverage and then plateau in the anodic sweep. However, instead, experiments find a decrease in carbonate at φ > -0.85 V. We model this phenomenon by applying a continuous V-shaped binding energy to carbonate in the form of:

$$\Delta E_{CO_3{}^{2-}} = \begin{cases} \Delta E_{PZC,CO_3{}^{2-}} + \alpha_{+,CO_3{}^{2-}}(-2)(\varphi + 0.9)\,, \varphi \geq \varphi_{Vpt} \\ \Delta E_{PZC,CO_3{}^{2-}} + \alpha_{-,CO_3{}^{2-}}(-2)(\varphi + 0.9)\,, \varphi < \varphi_{Vpt} \end{cases}$$

where φ_{Vpt} is the potential at which the vertex of the V-shaped binding energy exists and $\alpha_{+,CO_3{}^{2-}}$ and $\alpha_{-,CO_3{}^{2-}}$ modulate the slopes on either side of φ_{Vpt}. Fitting to this V-shaped binding energy to experiment (a) (Ar-saturated 0.25 M Na_2CO_3), we get $\varphi_{Vpt} = -0.8486\,V$. The fitted values for $\alpha_{+,CO_3{}^{2-}}$ and $\alpha_{-,CO_3{}^{2-}}$ are given in **Table** .

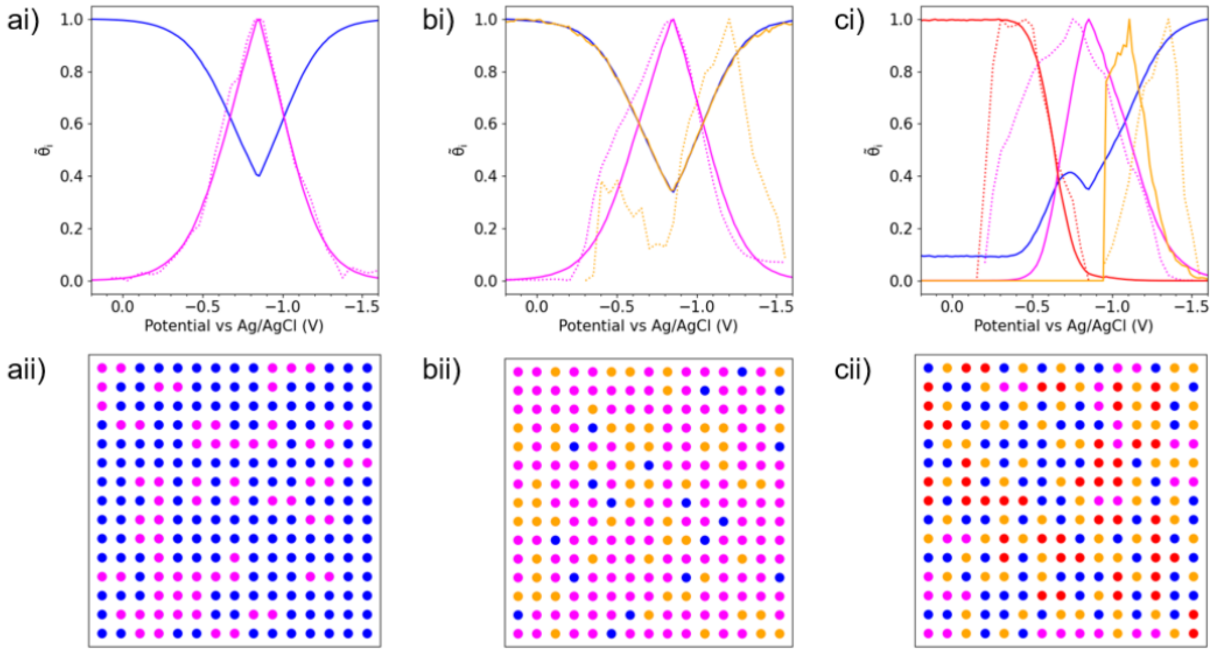

Figure S30. Monte Carlo simulation results for the competition adsorption.

Under the three conditions: **ai,ii)** $*H_2O$ and $*CO_3{}^{2-}$. SHINER measurement conditions (0.25 M Na_2CO_3 purged with Ar gas, pH = 11.95), **bi,ii)** $*H_2O$, $*CO_3{}^{2-}$, and *CO, SHINER measurement conditions (0.5 M $NaHCO_3$ saturated with CO gas, pH = 8.44), and **ci,ii)** $*H_2O$, $*CO_3{}^{2-}$, $*COO^-$, and *CO under CO_2RR conditions. SHINER measurement conditions (0.5 M $NaHCO_3$ saturated with CO_2 gas, pH = 7.2). **ai, bi, ci**) Comparisons of the simulated normalized surface coverage of each adsorbate and the measured normalized Raman intensity as a function of applied potential: $*H_2O$ (blue), $*CO_3{}^{2-}$ (pink), *CO (orange), and $*COO^-$ (red). **aii, bii, cii**) Examples of the

simulated surface population with lattice sites color-coded by the type of adsorbate occupying the site.

Table S3. Adsorbate Parameters for Monte Carlo simulations.

Adsorbate	q_i (e)	m_i (mols/kg water)			Number of Lattice Sites	Fitted $\Delta E_{PZC,i}$ (eV)	Literature $\Delta E_{PZC,i}$ (eV)	DFT $\Delta E_{PZC,i}$ (eV)	α_i
		(a)	(b)	(c)					
*H_2O	0	55.56			1	0	-0.6[a]	-0.00917	0
*CO_3^{2-}	-2	0.25	0.5	0.5	2	-0.2617		-5.17569	0.1065/-0.09669
*CO	0	0	0.0009639	0	1	-0.237	-0.73[b] (monolayer) -0.55[c] (single)	-0.48649	0
*COO^-	-1	0	0	0.0234	2	-0.24	-0.25[d]	-4.79459	0.35

The molalities are from the experimental conditions: a) C1 electrolyte (Ar-saturated 0.25 M Na_2CO_3), b) C3 electrolyte (CO-saturated 0.5 M $NaHCO_3$, and c) E1 electrolyte (CO_2-saturated 0.5 M $NaHCO_3$). The fit binding energies ($\Delta E_{PZC,i}$) at the potential of zero charge are shifted such that $\Delta E_{PZC,H2O}$= 0 eV. These fit binding energies are compared to reported binding energies (unsolvated) and our DFT-calculated binding energies in implicit solvent. Binding energies references: a: Zapol et al.[45]; b: Bauschlicher et al.[46] ; c: Gameel et al.[47]; d: Rasmussen et al.[48]

Figure S30 depicts examples of the three simulated lattice systems and their normalized surface coverages as a function of applied potential. Each adsorbate's surface coverage is normalized by that adsorbate's maximum surface coverage over the potential region scanned. The simulated normalized surface coverage for an adsorbate (solid line) is compared to the normalized Raman intensity associated with that adsorbate (dotted line). **Table** lists all adsorption parameters fit to the experimental results. While some DFT and literature values exist for adsorbate binding energies, we find that, given the simplicity of our model, fitting parameters

to experimental data offers a suitable test on the ability of competitive adsorption to elucidate trends in surface coverage.

The origin of decreasing binding energy of carbonate at more positive potentials is unclear, although we offer a number of possible reasons. The first and most likely reason is that carbonate adsorption is hindered by the slow Cu_2O to Cu reduction in the cathodic sweep, which may also include surface reconstruction. If this is the case, it would appear that the state of oxidation at the electrode surface affects carbonate adsorption at positive potentials more than it does carboxylate production or *CO adsorption. This is because both $*COO^-$ (**Figure ci**) and *CO (**Figure bi**) are both empirically present on the surface at potentials greater (more positive) than the potential at which adsorbed carbonate starts to decline. The reason for this discrepancy in effect may be due to binding sites heterogeneity where carbonate binds to sites that are more affected by the surface reconstruction. Another likely possibility is a gradual surface rearrangement of the carbonate-water adlayer that causes the detected decrease in carbonate coverage[49]. A second reason is that carbonate's desorption from the electrode in the anodic scan is induced by a potential-dependent change in pH. However, we argue that this may not be the full picture, since carbonate's Raman intensity vs. applied potential profile stays relatively unchanged across carbonate solutions with very different pHs (**Figure S30 ai, bi, ci**). A third possible reason is that carbonate is being competitively "kicked off" by a more strongly binding adsorbate. However, this also seems not likely because if there was a strong adsorbate, it would not only remove $*CO_3^{2-}$ but also $*COO^-$ and *CO at φ > -0.85, and yet one does detect $*COO^-$ and *CO at φ > -0.85. No such stronger adsorbate, outside of hydroxyls, has been detected in this potential region, but this explanation cannot be completely excluded if facet heterogeneity is a consideration. Our study suggests that, regardless of the source of the *ad hoc* V-shaped binding energy for carbonate, it is necessary to produce the two local maxima for *CO coverage in CO-saturated bicarbonate solution (**Figure S30 bi**).

In the simulation of **Figure S30 ci**, the $*COO^-$ to *CO reaction is modeled as the reversible kinetic pathway $*COO^- \rightleftharpoons *CO + *H_2O$ for $\varphi \leq \varphi_{CO}$, the onset potential of CO. Due

to this model, it is impossible to fully eliminate overlap between the potential range in which $*COO^-$ is on the surface and the potential range in which *CO is on the surface. At most, one can set a very negative φ_{CO} such that *CO appears when $*COO^-$ is minimal. In this model, φ_{CO} is -0.95 V. Consequently, the shape of *CO's coverage *vs.* applied potential curve is dictated by φ_{CO}, CO's charge (which matches water's), $*COO^-$'s binding energy, and competition for sites with $*CO_3^{2-}$. In the cathodic sweep, at φ_{CO}, *CO coverage increases, following the slope of $*H_2O$ since both are neutral. At around -1.15 V, *CO coverage maximizes and then begins to decrease, unlike water's coverage. This decrease is caused by the decreasing $*COO^-$ coverage due to $*COO^-$'s negative charge.

Figure S30 ci also suggests that competitive adsorption between $*COO^-$ and $*CO_3^{2-}$ may not fully explain the potential-dependent coverages we observe under CO_2 reduction conditions. With competitive adsorption, $*COO^-$ delays $*CO_3^{2-}$ onset to more cathodic potentials. However, in experiments, one observes $*COO^-$ and $*CO_3^{2-}$ co-adsorbing between -0.2 and -0.5 V. **Table** 's empirically fitted parameters indicates significantly weaker intrinsic binding energies ($\Delta E_{PZC,i}$) and potential-dependence of binding energies (α_i) than reported in literature and calculated with DFT. The binding energies at the potential of zero charge in our model may be weaker than those in DFT due to DFT not fully accounting for the partial desolvation of the adsorbate that is required to enter the inner Helmholtz layer. Both the intrinsic binding energy and potential-dependence are also influenced by lateral interactions between adsorbates and image charges. For CO adsorption, it is known that CO's binding energy increases with increasing surface coverage.[b] It also increases with increased coverage of OH[50–52]. For adsorbed anions like $*COO^-$ and $*CO_3^{2-}$, one might expect repulsion between species of like charge on the surface that causes lower binding energies at higher coverage. However, it is also known that anions are able to form compact and highly structured adlayers on the surface with H_2O, suggesting more nuanced interactions[53,54].

2.14 Additional control results

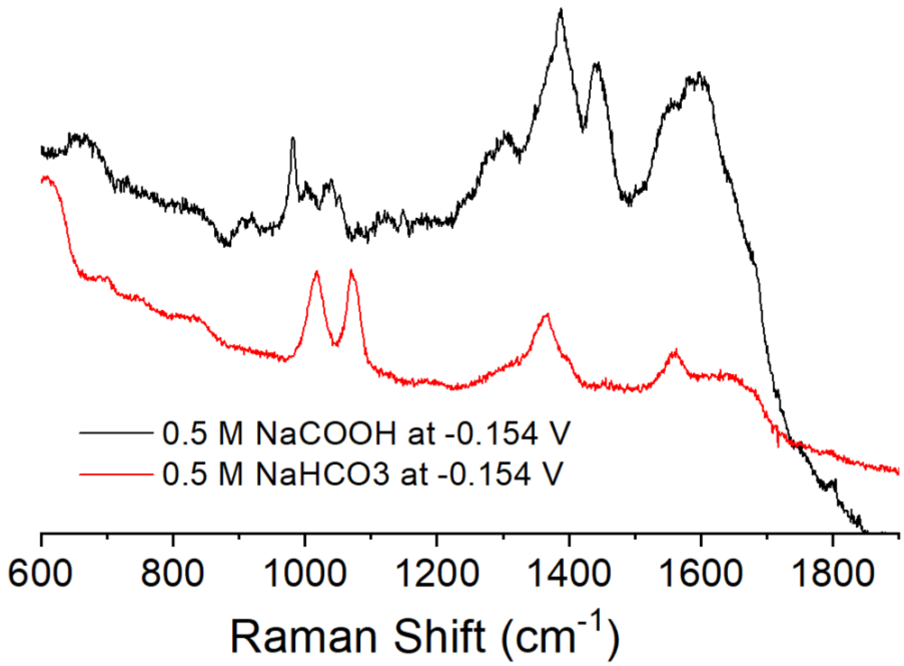

Figure S31. The SHINERS spectra of formate control (0.5 M NaCOOH)

The *COO⁻ intermediates can vary across different metal surfaces depending on their affinity toward *H and the specific M–COO⁻ binding configurations, thus there is possibilities of a *COO⁻ intermediate leading to formate production. To strictly rule this possibility out, we also did a control SHINERS measurement with Sodium formate solution on Cu electrode (control spectra of NaCOOH) to compare with our spectra under CO2RR.

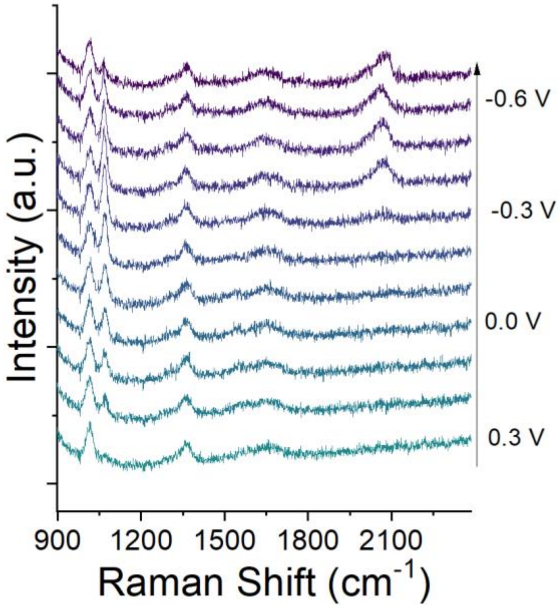

Figure S32. The SERS spectra of CO2RR on Cu bare electrodes under the same conditions.

The SERS spectra was obtained on the Cu electrode which was prepared under the same conditions compared with the electrodes used for this study. The signal-to-noise ratio is noticeable lower than the SHINERS spectra shown in the Figure 2. The low signal-to-noise ratio is mainly due to the low SERS activity for the bare untreated Cu surfaces.

3. References

(1) Li, J. F.; Huang, Y. F.; Ding, Y.; Yang, Z. L.; Li, S. B.; Zhou, X. S.; Fan, F. R.; Zhang, W.; Zhou, Z. Y.; Wu, D. Y.; Ren, B.; Wang, Z. L.; Tian, Z. Q. Shell-Isolated Nanoparticle-Enhanced Raman Spectroscopy. *Nature* **2010**, *464* (7287), 392–395. https://doi.org/10.1038/nature08907.

(2) Li, C.-Y.; Chen, M.; Liu, S.; Lu, X.; Meng, J.; Yan, J.; Abruña, H. D.; Feng, G.; Lian, T. Unconventional Interfacial Water Structure of Highly Concentrated Aqueous Electrolytes at Negative Electrode Polarizations. *Nat. Commun.* **2022**, *13* (1), 5330. https://doi.org/10.1038/s41467-022-33129-8.

(3) Wang, H.; Rus, E.; Abruña, H. D. New Double-Band-Electrode Channel Flow Differential Electrochemical Mass Spectrometry Cell: Application for Detecting Product Formation during Methanol Electrooxidation. *Anal. Chem.* **2010**, *82* (11), 4319–4324. https://doi.org/10.1021/ac100320a.

(4) Wang, H.; Abruña, H. D. Adsorbed Enolate as the Precursor for the C–C Bond Splitting during Ethanol Electrooxidation on Pt. *J. Am. Chem. Soc.* **2023**, *145* (11), 6330–6338. https://doi.org/10.1021/jacs.2c13401.

(5) Rudolph, W. W.; Irmer, G.; Königsberger, E. Speciation Studies in Aqueous HCO_3^-–CO_3^{2-} Solutions. A Combined Raman Spectroscopic and Thermodynamic Study. *Dalton Trans* **2008**, No. 7, 900–908. https://doi.org/10.1039/B713254A.

(6) Shao, F.; Wong, J. K.; Low, Q. H.; Iannuzzi, M.; Li, J.; Lan, J. In Situ Spectroelectrochemical Probing of CO Redox Landscape on Copper Single-Crystal Surfaces. *Proc. Natl. Acad. Sci.* **2022**, *119* (29), e2118166119. https://doi.org/10.1073/pnas.2118166119.

(7) Rudolph, W. W.; Fischer, D.; Irmer, G. Vibrational Spectroscopic Studies and Density Functional Theory Calculations of Speciation in the CO_2—Water System. *Appl. Spectrosc.* **2006**, *60* (2), 130–144. https://doi.org/10.1366/000370206776023421.

(8) Zosel, J.; Oelßner, W.; Decker, M.; Gerlach, G.; Guth, U. The Measurement of Dissolved and Gaseous Carbon Dioxide Concentration. *Meas. Sci. Technol.* **2011**, *22* (7), 072001. https://doi.org/10.1088/0957-0233/22/7/072001.

(9) Chernyshova, I. V.; Ponnurangam, S.; Somasundaran, P. Linking Interfacial Chemistry of CO2 to Surface Structures of Hydrated Metal Oxide Nanoparticles: Hematite. *Phys. Chem. Chem. Phys.* **2013**, *15* (18), 6953. https://doi.org/10.1039/c3cp44264k.

(10) Iwasita, T.; Rodes, A.; Pastor, E. Vibrational Spectroscopy of Carbonate Adsorbed on Pt(111) and Pt(110) Single-Crystal Electrodes. *J. Electroanal. Chem.* **1995**, *383* (1–2), 181–189. https://doi.org/10.1016/0022-0728(94)03708-B.

(11) Lu, X.; Zhu, C.; Wu, Z.; Xuan, J.; Francisco, J. S.; Wang, H. In Situ Observation of the pH Gradient near the Gas Diffusion Electrode of CO_2 Reduction in Alkaline Electrolyte. *J. Am. Chem. Soc.* **2020**, *142* (36), 15438–15444. https://doi.org/10.1021/jacs.0c06779.

(12) Zhu, S.; Jiang, B.; Cai, W.-B.; Shao, M. Direct Observation on Reaction Intermediates and the Role of Bicarbonate Anions in CO_2 Electrochemical Reduction Reaction on Cu Surfaces. *J. Am. Chem. Soc.* **2017**, *139* (44), 15664–15667. https://doi.org/10.1021/jacs.7b10462.

(13) Heyes, J.; Dunwell, M.; Xu, B. CO_2 Reduction on Cu at Low Overpotentials with Surface-Enhanced in Situ Spectroscopy. *J. Phys. Chem. C* **2016**, *120* (31), 17334–17341. https://doi.org/10.1021/acs.jpcc.6b03065.

(14) Moradzaman, M.; Mul, G. In Situ Raman Study of Potential-Dependent Surface Adsorbed Carbonate, CO, OH, and C Species on Cu Electrodes During Electrochemical Reduction of CO2. *ChemElectroChem* **2021**, *8* (8), 1478–1485. https://doi.org/10.1002/celc.202001598.

(15) Jiang, S.; D'Amario, L.; Dau, H. Copper Carbonate Hydroxide as Precursor of Interfacial CO in CO_2 Electroreduction. *ChemSusChem* **2022**, *15* (8). https://doi.org/10.1002/cssc.202102506.

(16) Firet, N. J.; Smith, W. A. Probing the Reaction Mechanism of CO_2 Electroreduction over Ag Films via Operando Infrared Spectroscopy. *ACS Catal.* **2017**, *7* (1), 606–612. https://doi.org/10.1021/acscatal.6b02382.

(17) Iijima, G.; Inomata, T.; Yamaguchi, H.; Ito, M.; Masuda, H. Role of a Hydroxide Layer on Cu Electrodes in Electrochemical CO_2 Reduction. *ACS Catal.* **2019**, *9* (7), 6305–6319. https://doi.org/10.1021/acscatal.9b00896.

(18) Gunathunge, C. M.; Li, X.; Li, J.; Hicks, R. P.; Ovalle, V. J.; Waegele, M. M. Spectroscopic Observation of Reversible Surface Reconstruction of Copper Electrodes under CO_2 Reduction. *J. Phys. Chem. C* **2017**, *121* (22), 12337–12344. https://doi.org/10.1021/acs.jpcc.7b03910.

(19) Zhan, C.; Dattila, F.; Rettenmaier, C.; Bergmann, A.; Kühl, S.; García-Muelas, R.; López, N.; Cuenya, B. R. Revealing the CO Coverage-Driven C–C Coupling Mechanism for Electrochemical CO_2 Reduction on Cu_2O Nanocubes *via Operando* Raman Spectroscopy. *ACS Catal.* **2021**, *11* (13), 7694–7701. https://doi.org/10.1021/acscatal.1c01478.

(20) Niaura, G. Surface-Enhanced Raman Spectroscopic Observation of Two Kinds of Adsorbed OH^- Ions at Copper Electrode. *Electrochimica Acta* **2000**, *45* (21), 3507–3519. https://doi.org/10.1016/S0013-4686(00)00434-5.

(21) Bodappa, N.; Su, M.; Zhao, Y.; Le, J.-B.; Yang, W.-M.; Radjenovic, P.; Dong, J.-C.; Cheng, J.; Tian, Z.-Q.; Li, J.-F. Early Stages of Electrochemical Oxidation of Cu(111) and Polycrystalline Cu Surfaces Revealed by *in Situ* Raman Spectroscopy. *J. Am. Chem. Soc.* **2019**, *141* (31), 12192–12196. https://doi.org/10.1021/jacs.9b04638.

(22) Chernyshova, I. V.; Somasundaran, P.; Ponnurangam, S. On the Origin of the Elusive First Intermediate of CO_2 Electroreduction. *Proc. Natl. Acad. Sci.* **2018**, 115 (40) E9261-E9270. https://doi.org/10.1073/pnas.1802256115.

(23) Pfisterer, J. H. K.; Zhumaev, U. E.; Cheuquepan, W.; Feliu, J. M.; Domke, K. F. Stark Effect or Coverage Dependence? Disentangling the EC-SEIRAS Vibrational Shift of Sulfate on Au(111). *J. Chem. Phys.* **2019**, *150* (4), 041709. https://doi.org/10.1063/1.5047941.

(24) Jiang, S.; Klingan, K.; Pasquini, C.; Dau, H. New Aspects of Operando Raman Spectroscopy Applied to Electrochemical CO_2 Reduction on Cu Foams. *J. Chem. Phys.* **2019**, *150* (4), 041718. https://doi.org/10.1063/1.5054109.

(25) Kloprogge, J. T.; Wharton, D.; Hickey, L.; Frost, R. L. Infrared and Raman Study of Interlayer Anions CO_3^{2-}, NO_3^-, SO_4^{2-} and ClO_4^- in Mg/Al-Hydrotalcite. *Am. Mineral.* **2002**, *87* (5–6), 623–629. https://doi.org/10.2138/am-2002-5-604.

(26) Arihara, K.; Kitamura, F.; Ohsaka, T.; Tokuda, K. Characterization of the Adsorption State of Carbonate Ions at the Au(111) Electrode Surface Using in Situ IRAS. *J. Electroanal. Chem.* **2001**, *510* (1–2), 128–135. https://doi.org/10.1016/S0022-0728(01)00498-3.

(27) Eilert, A.; Roberts, F. S.; Friebel, D.; Nilsson, A. Formation of Copper Catalysts for CO_2 Reduction with High Ethylene/Methane Product Ratio Investigated with In Situ X-Ray Absorption Spectroscopy. *J. Phys. Chem. Lett.* **2016**, *7* (8), 1466–1470. https://doi.org/10.1021/acs.jpclett.6b00367.

(28) Spodaryk, M.; Zhao, K.; Zhang, J.; Oveisi, E.; Züttel, A. The Role of Malachite Nanorods for the Electrochemical Reduction of CO2 to C2 Hydrocarbons. *Electrochimica Acta* **2019**, *297*, 55–60. https://doi.org/10.1016/j.electacta.2018.11.124.

(29) Henckel, D. A.; Counihan, M. J.; Holmes, H. E.; Chen, X.; Nwabara, U. O.; Verma, S.; Rodríguez-López, J.; Kenis, P. J. A.; Gewirth, A. A. Potential Dependence of the Local pH in a CO_2 Reduction Electrolyzer. *ACS Catal.* **2021**, *11* (1), 255–263. https://doi.org/10.1021/acscatal.0c04297.

(30) Dunwell, M.; Yang, X.; Setzler, B. P.; Anibal, J.; Yan, Y.; Xu, B. Examination of Near-Electrode Concentration Gradients and Kinetic Impacts on the Electrochemical Reduction of CO_2 Using Surface-Enhanced Infrared Spectroscopy. *ACS Catal.* **2018**, *8* (5), 3999–4008. https://doi.org/10.1021/acscatal.8b01032.

(31) Wuttig, A.; Ryu, J.; Surendranath, Y. Electrolyte Competition Controls Surface Binding of CO Intermediates to CO_2 Reduction Catalysts. *J. Phys. Chem. C* **2021**, *125* (31), 17042–17050. https://doi.org/10.1021/acs.jpcc.1c04337.

(32) Hori, Y.; Koga, O.; Watanabe, Y.; Matsuo, T. FTIR Measurements of Charge Displacement Adsorption of CO on Poly- and Single Crystal (100) of Cu Electrodes. *Electrochimica Acta* **1998**, *44* (8–9), 1389–1395. https://doi.org/10.1016/S0013-4686(98)00261-8.

(33) de Ruiter, J.; An, H.; Wu, L.; Gijsberg, Z.; Yang, S.; Hartman, T.; Weckhuysen, B. M.; van der Stam, W. Probing the Dynamics of Low-Overpotential CO_2-to-CO Activation on Copper Electrodes with Time-Resolved Raman Spectroscopy. *J. Am. Chem. Soc.* **2022**, *144* (33), 15047–15058. jacs.2c03172. https://doi.org/10.1021/jacs.2c03172.

(34) Kimura, K. W.; Casebolt, R.; Cimada DaSilva, J.; Kauffman, E.; Kim, J.; Dunbar, T. A.; Pollock, C. J.; Suntivich, J.; Hanrath, T. Selective Electrochemical CO_2 Reduction during Pulsed Potential Stems from Dynamic Interface. *ACS Catal.* **2020**, *10* (15), 8632–8639. https://doi.org/10.1021/acscatal.0c02630.

(35) Zhao, Y.; Chang, X.; Malkani, A. S.; Yang, X.; Thompson, L.; Jiao, F.; Xu, B. Speciation of Cu Surfaces During the Electrochemical CO Reduction Reaction. *J. Am. Chem. Soc.* **2020,** *142* (21) 9735–9743. https://doi.org/10.1021/jacs.0c02354.

(36) Chang, X.; Zhao, Y.; Xu, B. pH Dependence of Cu Surface Speciation in the Electrochemical CO Reduction Reaction. *ACS Catal.* **2020**, *10* (23), 13737–13747. https://doi.org/10.1021/acscatal.0c03108.
(37) Kim, Y.; Park, S.; Shin, S.-J.; Choi, W.; Min, B. K.; Kim, H.; Kim, W.; Hwang, Y. J. Time-Resolved Observation of C–C Coupling Intermediates on Cu Electrodes for Selective Electrochemical CO_2 Reduction. *Energy Environ. Sci.* **2020**, *13* (11), 4301–4311. https://doi.org/10.1039/D0EE01690J.
(38) Pérez-Gallent, E.; Marcandalli, G.; Figueiredo, M. C.; Calle-Vallejo, F.; Koper, M. T. M. Structure- and Potential-Dependent Cation Effects on CO Reduction at Copper Single-Crystal Electrodes. *J. Am. Chem. Soc.* **2017**, *139* (45), 16412–16419. https://doi.org/10.1021/jacs.7b10142.
(39) Pérez-Gallent, E.; Figueiredo, M. C.; Calle-Vallejo, F.; Koper, M. T. M. Spectroscopic Observation of a Hydrogenated CO Dimer Intermediate During CO Reduction on Cu(100) Electrodes. *Angew. Chem.* **2017**, *129* (13), 3675–3678. https://doi.org/10.1002/ange.201700580.
(40) Łukomska, A.; Sobkowski, J. Potential of Zero Charge of Monocrystalline Copper Electrodes in Perchlorate Solutions. *J. Electroanal. Chem.* **2004**, *567* (1), 95–102. https://doi.org/10.1016/j.jelechem.2003.11.063.
(41) Gileadi, E.; Argade, S. D.; Bockris, J. O. The Potential of Zero Charge of Platinum and Its pH Dependence. *J. Phys. Chem.* **1966**, *70* (6), 2044–2046. https://doi.org/10.1021/j100878a501.
(42) Auer, A.; Ding, X.; Bandarenka, A. S.; Kunze-Liebhäuser, J. The Potential of Zero Charge and the Electrochemical Interface Structure of Cu(111) in Alkaline Solutions. *J. Phys. Chem. C* **2021**, *125* (9), 5020–5028. https://doi.org/10.1021/acs.jpcc.0c09289.
(43) Hori, Y.; Murata, A.; Takahashi, R. Formation of Hydrocarbons in the Electrochemical Reduction of Carbon Dioxide at a Copper Electrode in Aqueous Solution. *J. Chem. Soc. Faraday Trans. 1 Phys. Chem. Condens. Phases* **1989**, *85* (8), 2309. https://doi.org/10.1039/f19898502309.
(44) Kuhl, K. P.; Cave, E. R.; Abram, D. N.; Jaramillo, T. F. New Insights into the Electrochemical Reduction of Carbon Dioxide on Metallic Copper Surfaces. *Energy Environ. Sci.* **2012**, *5* (5), 7050. https://doi.org/10.1039/c2ee21234j.
(45) Zapol, P.; Naleway, C. A.; Deutsch, P. W.; Curtiss, L. A. Electronic Structure Studies of the Interaction of Water with a Cu(100) Surface. In *Solid-Liquid Interface Theory*; ACS Symposium Series; American Chemical Society, 2001; Vol. 789, pp 3–9. https://doi.org/10.1021/bk-2001-0789.ch001.
(46) Bauschlicher, C. W. A Theoretical Study of CO/Cu(100). *J. Chem. Phys.* **1994**, *101* (4), 3250–3254. https://doi.org/10.1063/1.467572.
(47) Gameel, K. M.; Sharafeldin, I. M.; Abourayya, A. U.; Biby, A. H.; Allam, N. K. Unveiling CO Adsorption on Cu Surfaces: New Insights from Molecular Orbital Principles. *Phys. Chem. Chem. Phys.* **2018**, *20* (40), 25892–25900. https://doi.org/10.1039/C8CP04253E.

(48) Rasmussen, P. B.; Taylor, P. A.; Chorkendorff, I. The Interaction of Carbon Dioxide with Cu(100). *Surf. Sci.* **1992**, *269–270*, 352–359. https://doi.org/10.1016/0039-6028(92)91274-F.

(49) Amirbeigiarab, R.; Bagger, A.; Tian, J.; Rossmeisl, J.; Magnussen, O. M. Structure of the (Bi)Carbonate Adlayer on Cu(100) Electrodes. *Angew. Chem. Int. Ed.* **2022**, *61* (46). https://doi.org/10.1002/anie.202211360.

(50) Tiwari, A.; Heenen, H. H.; Bjørnlund, A. S.; Hochfilzer, D.; Chan, K.; Horch, S. Electrochemical Oxidation of CO on Cu Single Crystals under Alkaline Conditions. *ACS Energy Lett.* **2020**, *5* (11), 3437–3442. https://doi.org/10.1021/acsenergylett.0c01751.

(51) Iijima, G.; Inomata, T.; Yamaguchi, H.; Ito, M.; Masuda, H. Role of a Hydroxide Layer on Cu Electrodes in Electrochemical CO_2 Reduction. *ACS Catal.* **2019**, *9* (7), 6305–6319. https://doi.org/10.1021/acscatal.9b00896.

(52) Dinh, C.-T.; Burdyny, T.; Kibria, M. G.; Seifitokaldani, A.; Gabardo, C. M.; García de Arquer, F. P.; Kiani, A.; Edwards, J. P.; De Luna, P.; Bushuyev, O. S.; Zou, C.; Quintero-Bermudez, R.; Pang, Y.; Sinton, D.; Sargent, E. H. CO_2 Electroreduction to Ethylene via Hydroxide-Mediated Copper Catalysis at an Abrupt Interface. *Science* **2018**, *360* (6390), 783–787. https://doi.org/10.1126/science.aas9100.

(53) Magnussen, O. M. Ordered Anion Adlayers on Metal Electrode Surfaces. *Chem. Rev.* **2002**, *102* (3), 679–726. https://doi.org/10.1021/cr000069p.

(54) Magnussen, O. M.; Groß, A. Toward an Atomic-Scale Understanding of Electrochemical Interface Structure and Dynamics. *J. Am. Chem. Soc.* **2019**, *141* (12), 4777–4790. https://doi.org/10.1021/jacs.8b13188.
